# Parameter extraction of PV models under varying meteorological conditions using a modified electric eel foraging optimization algorithm

**DOI:** 10.1038/s41598-025-98270-y

**Published:** 2025-06-02

**Authors:** Hadeer Khalifa, Mohamed Ebeed, Gaber Magdy, Sherif A. Khaleel, Mohamed I. Shehata, Moataz M. Salah, Francisco Jurado, Hossam Hassan Ali

**Affiliations:** 1 Department of Electronics & Communications Engineering, College of Engineering and Technology, Arab Academy for Science, Technology and Maritime Transport, Aswan, 81511 Egypt; 2 Department of Electrical Engineering, Faculty of Engineering, Sohag University, Sohag, 82524 Egypt; 3 Department of Electrical Engineering, EPS Linares, University of Jaén, 23700 Jaén, Spain; 4 Department of Electrical Engineering, Faculty of Energy Engineering, Aswan University, Aswan, 81528 Egypt; 5 Faculty of Engineering, King Salman International University, El-Tor, South Sinai 46511 Egypt; 6 Electrical Department, Faculty of Technology and Education, Sohag University, Sohag, 82524 Egypt

**Keywords:** Solar PV system, Parameter extraction, PV modules, Modified electric eel foraging optimization (MEEFO), Electrical and electronic engineering, Solar cells

## Abstract

The dependence on photovoltaic (PV) solar systems has increased dramatically to cover the increasing progress of world energy demand. Therefore, accurately specifying the parameters of PV modules is essential for evaluating the behavior and impact of integrating PV systems into electrical systems. In this context, a modified electric eel foraging optimization (MEEFO) is suggested for determining the parameters of solar PV modules. The proposed technique incorporates three improvement strategies: the fitness distance balance (FDB) strategy, fractional-order calculus (FOC), and quasiopposition-based learning (QOBL). These strategies enhance both exploitation and exploration capabilities while helping to prevent local optimization and premature convergence commonly observed in traditional EEFO. First, the proposed MEEFO is evaluated via two benchmark functions, including the basic and CEC 2019 benchmark functions. The results are then compared with those of other novel methods in terms of accuracy, convergence characteristics, and overall performance. The suggested MMEFO is then employed to identify the parameters for the single, double, and triple diode models of various PV cells/modules, including R.T.C. France, PVM752, STM6-40/36, PWP-201, and STP6-120/36. In addition, various meteorological data, such as changes in radiation and temperature, exist. The simulation findings demonstrate that MEEFO outperforms other techniques and is a reliable and superior method for accurately estimating PV module parameters. The application of MEEFO yields the lowest root mean square error (RMSE) values for the considered single, double, and triple diode models of R.T.C. France. Similarly, for STP6-120/36, the RMSE values are 1.660060E−02, 1.66006E−02, and 1.66089E−02, respectively. Additionally, for PWP-20, the RMSE values are 2.425075E−03, 2.42511E−03, and 2.42510E−03, respectively.

**Supplementary Information:**

The online version contains supplementary material available at 10.1038/s41598-025-98270-y.

## Introduction

Renewable energy systems have attracted increasing attention in recent years because of the gradual depletion of fossil fuels such as oil, coal, and natural gas. Solar energy, as one of the most abundant and inexhaustible resources, offers a promising alternative to conventional fossil fuels, which are major contributors to environmental pollution and greenhouse gas emissions^1–3^. Solar cells, which convert sunlight directly into electrical energy via photovoltaic (PV) technology, offer a clean, sustainable, and efficient solution to these challenges^4^. PV systems have been implemented in a range of applications across various sectors and can be categorized into five distinct types: grid-connected PV systems, standalone or isolated PV systems, hybrid PV generation systems, solar power plants, and PV cells integrated into diverse products and applications. These applications span a wide range of systems, including solar roofs, irrigation networks, electrical equipment, electric vehicles, traffic light signals, and guiding robots^5–8^. The variability in climate conditions across different regions, seasons, and even throughout a single day highlights the urgent need for accurate determination of PV model parameters to effectively optimize power control and maximize efficiency. The modelling of PV solar cells is conducted through a series of steps: choosing the appropriate equivalent circuit configuration, deriving the equations for the selected model, and optimizing the equivalent circuit parameters. To enhance the overall performance of solar cells, it is essential to identify the optimal parameter values by selecting the appropriate equivalent circuit model and analysing the (I-V) and (P-V) curves. These curves are influenced by several factors, including ambient temperature, incident solar irradiance, and the particular equivalent circuit configuration used in the PV model^9,10^. Given the widespread use of PV modules across various applications, significant advancements are required to ensure the optimal extraction of unknown parameters. These parameters vary on the basis of the mathematical model of the PV system, which can be categorized into a single-diode model (SDM) with five unknown parameters, a double-diode model (DDM) that requires the optimization of seven parameters, and a triple-diode model (TDM), which includes nine^11^.

The parameter estimation methods for the PV model can be divided into two categories: analytical methods and numerical methods. Analytical methods involve extracting PV parameters by solving the mathematical equations associated with PV systems^12^. This method applies only to the SDM because it consumes less time and data, making it less suitable for more complex DDMs and TDMs. The numerical methods involve mathematical programming and metaheuristic (MH) techniques for the PV parameter extraction process. The mathematical programming approach is highly sensitive to initial conditions and imposes stringent requirements on models, including conditions such as convexity and differentiability^13–15^. The mathematical technique is considered less accurate because of the nonlinear nature of the PV parameter extraction process. MH techniques are regarded as effective methods for extracting PV parameters because of their flexibility in terms of computation time and convergence. The MH approaches employ iterative methods, including Levenberg–Marquardt, Newton–Raphson, and linear identification, to obtain precise results. However, initial values greatly influence their effectiveness, and they can be time intensive when searching for the global solution^16–18^. Employing heuristic-based modern approaches is among the most promising solutions for tackling complex optimization problems. However, selecting the optimal technique can be difficult because of factors such as nonlinearity, multimodality, and other inherent characteristics of the optimization problem. The no-free-lunch theorem states that no single optimization method can reliably find the global optimum in every optimization scenario^19^. Consequently, researchers have utilized a range of MH optimization techniques to find the unknown parameters of PV models. These MH algorithms are commonly categorized based on their evolutionary principles, natural processes, human-inspired techniques, or biological foundations. The genetic algorithms^20^ and differential evolution algorithms^21^ are special cases of evolution-based algorithms. Using the statistical root-mean-square error (RMSE) to assess the variation between the actual and estimated output currents of a solar PV cell, MH approaches are applied to find the parameters of the SDM, DDM, and TDM^22^.

Throughout the past few years, researchers have used different MH optimization algorithms to address the challenge of estimating PV parameters. These algorithms can be categorized into four groups: evolution-based algorithms, swarm-based algorithms, mathematics-based algorithms, and physics-based algorithms^4^. Firstly, for evolution-based algorithms, various research topics are identified across different PV parameter determination models, including the SDM, DDM, and TDM. In^23^, a numerical technique utilizing genetic algorithms (GAs) was used to find the electrical parameters of solar PV cells and modules. The gain-sharing knowledge algorithm (GSKA), introduced in^24^, was employed alongside the GA optimization technique to develop the SDM, DDM, and PV panels. The backtracking search algorithm (BSA), mentioned in^25^, is utilized to address the parameter estimation problem of PV systems. Second, the swarm-based algorithms, which are considered the most suggested MH optimization algorithms, are among the different algorithms. Particle swarm optimization (PSO)^26^, competitive swarm optimization (CSO)^27^, Harris hawks optimization (HHO)^28^, cuckoo search optimization (CSO)^29,30^, and gray wolf optimization (GWO)^31^ have been introduced to estimate PV module parameters via various equivalent circuit models, including the SDM, DDM and TDM. The butterfly optimization algorithm (BOA) has been utilized to identify the parameters of the SDM and DDM circuits via experimental data from solar PV panels^32^. The shuffled frog leading algorithm (SFLA)^33^, pigeon-inspired optimization (PIO)^34^, northern goshawk optimization algorithm (NGOA)^35^, and robust niching chimp optimization (RNCO)^36^ have been used to determine the parameters of various PV cells/modules under different operating conditions. Third, the next category of MH techniques is mathematics-based algorithms. For example, the sine cosine algorithm (SCA) has been employed in various research papers, including^37^, to find the optimal parameters of PV-generating units. The JAYA algorithm^38^, transient search optimization (TSO)^39^, and spherical evolution algorithm (SEA)^40^ have been utilized to choose the parameters of three models of PV cells. Additionally, a hybrid sine‒cosine algorithm (SCA) combined with differential gradient-based optimization (GBO) was proposed for estimating PV parameters^41^. Finally, the physics-based MH algorithm represents the final category of MH algorithms. This approach has received relatively limited attention within the research community, as highlighted by studies such as the stochastic fractal search (SFS)^42^, simulated annealing (SA)^43^, and the equilibrium optimizer algorithm (EOA)^44^.

Despite their numerous advantages, MH approaches are not without limitations. One notable drawback is their susceptibility to becoming trapped in local minima, particularly when they are applied to high-dimensional problems, such as complex PV models. Due to the limitations of traditional MH techniques, several advanced methods have been developed and applied to improve the estimation of PV parameters.

One such method is the electric eel foraging optimization (EEFO) that’s introduced in different applications^45–47^. The EEFO has some limitations that can be avoided by a new modified version of the original algorithm MEEFO. modified electric eel foraging optimization (MEEFO), which is introduced in this paper. The contributions of this research can be summarized as follows:

- A new MEEFO based on three strategies, FDB, FOC, and QOBL, is proposed to enhance the search pattern of the conventional EEFO.
- MEEFO can be applied to accurately find the parameters of PV modules such as the SDM, DDM, and TDM.
- The validation, efficiency, and performance of MEEFO are verified via standard test suites and the CEC 2019 test suite.
- A comprehensive comparison is presented between MEEFO and Dung beetle optimize (BDO)^48^, the zebra optimization algorithm (ZOA)^49^, the African vultures optimization algorithm (AVOA)^50^, Harris hawks optimization (HHO)^51^, the sand cat search optimizer (SCSO)^52^, and the traditional EEFO.

The remaining sections of the paper are structured as follows: the problem statement is depicted in “Problem statement section. The concept and mathematical representation of the basic EEFO are presented in “Electric EEL foraging optimization algorithm” section. The proposed MEEFO is demonstrated in “Modified electric eel foraging optimization algorithm” section. The simulation findings and corresponding discussion are presented in “Simulation validation and analysis” section. The paper’s conclusions are outlined in the section.

## Problem statement

This section initially describes the mathematical formulas of the three PV models: the SDM, DDM, and TDM. It then introduces the cost function used for parameter identification in these PV models.

### Single diode model (SDM)

**Fig. 1:**
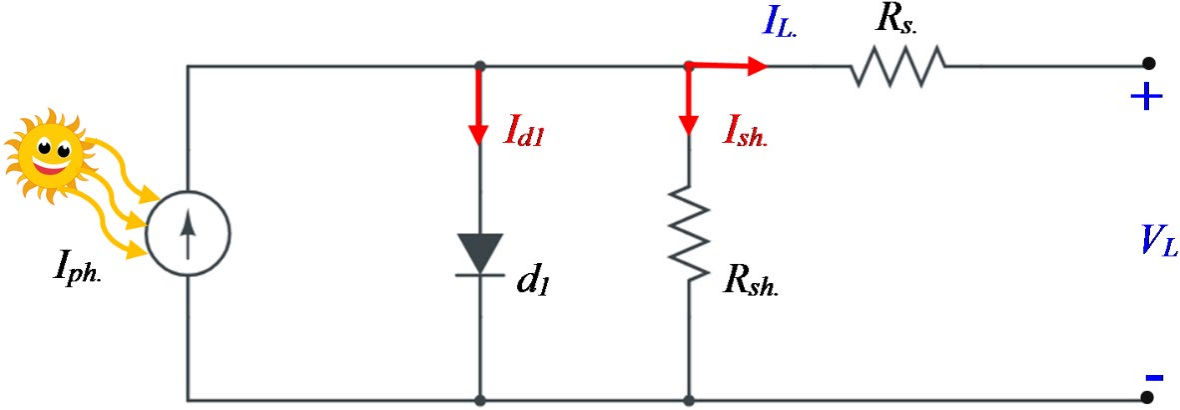
The equivalent circuit of SDM.

The SDM is an ideal choice for simulating the static characteristics of solar cells. As illustrated in Fig. 1, this model includes several parameters: a series resistance (*R*_*s*_), a shunt resistance (*R*_*sh*_), a rectifying diode (d), and a current source (*I*_*ph*_), which represents the photogenerated current from the solar irradiance (G). Kirchhoff’s current law (KCL) can be used for analyzing the output current (*I*_*L*_), as provided in Eq. (1)^4^.

1 $$\:{I}_{L}={I}_{ph}-{I}_{d1}-{I}_{sh}$$

where $\:{I}_{sh\:}$ represents the shunt resistance current and where $\:{I}_{d1}\:$ represents the current passing through the diode, which is calculated via the Shockley diode Eq. (2) listed below:

2 $$\:{I}_{d1}={I}_{R}\left[exp\left(\frac{e\left({V}_{L}+{I}_{L}{R}_{s}\right)}{\alpha\:KT}\right)-1\right]$$

where $\:{I}_{R}\:$ is defined as the reverse saturation current of the diode, $\:e\:$ represents the charge of the electron with a value of $\:(1.602\times\:{10}^{-19}C)$, $\:\alpha\:\:$ is defined as the ideality factor of the diode, $\:{V}_{L}\:$ represents the output voltage, $\:K$ is defined as the Boltzmann constant $\:(1.380\times\:{10}^{-23}J/K)$, and $\:T\:$ represents the temperature in Kelvin. Kirchhoff’s voltage law (KVL) can be applied in the equivalent circuit model shown in Fig. 1 to determine the current passing through the shunt resistance (*R*_*sh*_), and the value is as in Eq. (3).

3 $$\:{I}_{sh}=\frac{{V}_{L}+{I}_{L}{R}_{s}}{{R}_{sh}}$$

Based on Eqs. (2 and 3), Eq. (1) can be rewritten as:

4 $$\:{I}_{L}={I}_{ph}-{I}_{R}\left[exp\left(\frac{e\left({V}_{L}+{I}_{L}{R}_{s}\right)}{\alpha\:KT}\right)-1\right]-\frac{{V}_{L}+{I}_{L}{R}_{s}}{{R}_{sh}}$$

As indicated in Eq. (4), there are five unknown parameters, making the output current of the SDM a function of the following parameters:

5 $$\:f\left({x}_{1}\right)=\left[{I}_{ph},{I}_{R},{R}_{s},{R}_{sh},\alpha\:\:\:\right]$$

### Double diode model (DDM)

**Fig. 2:**
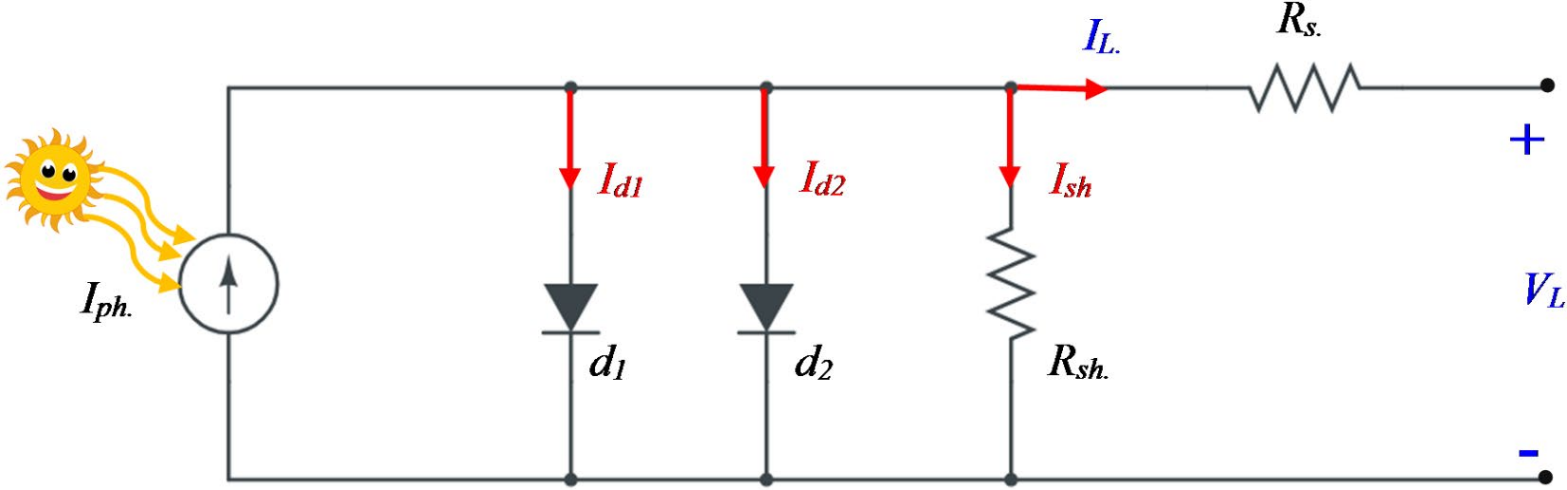
The equivalent circuit of the DDM.

The DDM in solar cells is introduced to improve the cell’s performance by minimizing the transmission losses that come from carrier and surface recombination in the depletion region. The model shown in Fig. 2 includes several parameters: a series resistance (*R*_*s*_), a shunt resistance (*R*_*sh*_), rectifying diodes (d1 and d2), and a current source (*I*_*ph*_), which represents the photogenerated current resulting from solar irradiance (G). According to the KCL, the output current (*I*_*L*_) can be determined via Eq. (6):

6 $$\:{I}_{L}={I}_{ph}-{I}_{d1}-{I}_{d2}{-I}_{sh}$$

where $\:{I}_{d1}\:,\:{I}_{d2}\:$ are the currents of the first and second diodes, respectively, which can be obtained via the Shockley diode equation and can be written as:

7 $$\:{I}_{d1}={I}_{R1}\left[exp\left(\frac{e\left({V}_{L}+{I}_{L}{R}_{s}\right)}{{\alpha\:}_{1}KT}\right)-1\right]$$

8 $$\:{I}_{d2}={I}_{R2}\left[exp\left(\frac{e\left({V}_{L}+{I}_{L}{R}_{s}\right)}{{\alpha\:}_{2}KT}\right)-1\right]$$

Equations (3), (6), (7), and (8) can be written as:

9 $$\:{I}_{L}={I}_{ph}-{I}_{R1}\left[exp\left(\frac{e\left({V}_{L}+{I}_{L}{R}_{s}\right)}{{\alpha\:}_{1}KT}\right)-1\right]-{I}_{R2}\left[exp\left(\frac{e\left({V}_{L}+{I}_{L}{R}_{s}\right)}{{\alpha\:}_{2}KT}\right)-1\right]-\frac{{V}_{L}+{I}_{L}{R}_{s}}{{R}_{sh}}$$

where $\:{I}_{R1},\:{I}_{R2},\:and\:{I}_{ph}$ are the reverse saturation currents of the two diodes and the photogenerated current, respectively. $\:{\alpha\:}_{1}\:and\:{\alpha\:}_{2}$ are the optimal factors of the two diodes. This model has seven unknown parameters that need to be calculated, which are expressed as a function in Eq. (10).

10 $$\:f\left({x}_{2}\right)=\left[{I}_{ph},{I}_{R1},{I}_{R2},{R}_{s},{R}_{sh},{\alpha\:}_{1},{\alpha\:}_{2}\:\:\right]$$

### Trible diode model (TDM)

**Fig. 3:**
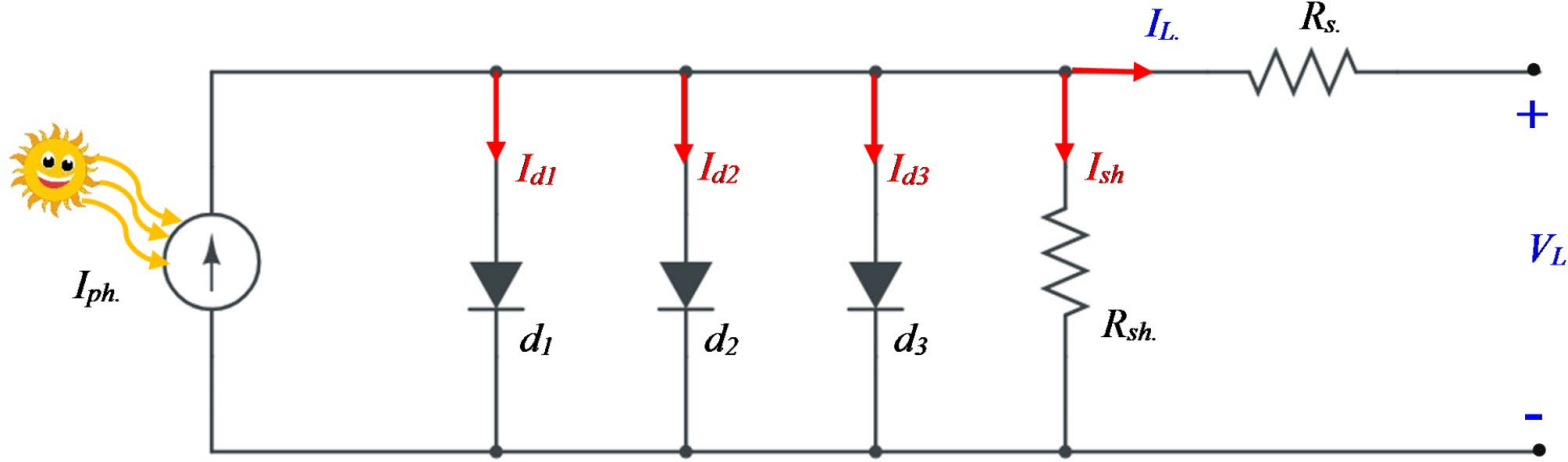
The equivalent circuit of TDM.

The Trible diode Model (TDM) of the PV system is presented to account for the effects of leakage current and grain boundaries. Figure 3 shows the equivalent circuit diagram of the TDM, and the formulation for generated current usage is given in Eqs. (11) and (12).

11 $$\:{I}_{L}={I}_{ph}-{I}_{d1}-{I}_{d2}-{I}_{d3}{-I}_{sh}$$

12 $$\:{I}_{L}={I}_{ph}-{I}_{R1}\left[exp\left(\frac{e\left({V}_{L}+{I}_{L}{R}_{s}\right)}{{\alpha\:}_{1}KT}\right)-1\right]-{I}_{R2}\left[exp\left(\frac{e\left({V}_{L}+{I}_{L}{R}_{s}\right)}{{\alpha\:}_{2}KT}\right)-1\right]-{I}_{R3}\left[exp\left(\frac{e\left({V}_{L}+{I}_{L}{R}_{s}\right)}{{\alpha\:}_{3}KT}\right)-1\right]-\frac{{V}_{L}+{I}_{L}{R}_{s}}{{R}_{sh}}$$

This model includes nine parameters that must be calculated, which are expressed as a function in Eq. (13).

13 $$\:f\left({x}_{2}\right)=\left[{I}_{ph},{I}_{R1},{I}_{R2},{I}_{R3},\:{R}_{s},{R}_{sh},{\alpha\:}_{1},{\alpha\:}_{2},{\alpha\:}_{3}\:\:\right]$$

### Objective function

To determine the unknown parameters of the PV models, the root mean square error (RMSE) between the measured current (*I*_*M*_) and the simulated current (*I*_*S*_) is commonly used as the objective function^32^. This can be determined as:

14 $$\:{F}_{RMSE}=\sqrt{\frac{1}{{N}_{data}}\sum\:_{i=1}^{{N}_{data}}{f}_{L}{\left({I}_{L},{V}_{L},x\right)}^{2}}=\sqrt{\frac{1}{{N}_{data}}\sum\:_{i=1}^{{N}_{data}}{\left({I}_{M}-{I}_{S}\right)}^{2}}$$

where the experimental data’s number is defined as $\:{N}_{data}$, $\:{f}_{L}$ represents the function of the PV model, and the measured and simulated currents can be referred to as the *I*_*M*_ and *I*_*S*_, respectively. According to Eq. (24), calculating the unknown parameters of the PV modules involves finding the optimal value of the $\:\left(x\right)$ corresponding minimum root mean square error. This value can vary depending on the selected PV model.

For the SDM, $\:{f}_{L}\left({I}_{L},{V}_{L},x\right)$ and $\:\left(x\right)$ can be expressed as:

15 $$\:\left\{\begin{array}{c}{f}_{L}\left({I}_{L},{V}_{L},x\right)={I}_{ph}-{I}_{R}\left[exp\left(\frac{e\left({V}_{L}+{I}_{L}{R}_{s}\right)}{\alpha\:KT}\right)-1\right]-\frac{{V}_{L}+{I}_{L}{R}_{s}}{{R}_{sh}}-{I}_{L}\\\:x=\left[{I}_{ph},{I}_{R},{R}_{s},{R}_{sh},\alpha\:\:\:\right]\end{array}\right.$$

For the DDM, $\:{f}_{L}\left({I}_{L},{V}_{L},x\right)$ and $\:\left(x\right)$ can be expressed as:

16 $$\:\left\{\begin{array}{c}{f}_{L}\left({I}_{L},{V}_{L},x\right)={I}_{ph}-{I}_{R1}\left[exp\left(\frac{e\left({V}_{L}+{I}_{L}{R}_{s}\right)}{{\alpha\:}_{1}KT}\right)-1\right]-{I}_{R2}\left[exp\left(\frac{e\left({V}_{L}+{I}_{L}{R}_{s}\right)}{{\alpha\:}_{2}KT}\right)-1\right]-\frac{{V}_{L}+{I}_{L}{R}_{s}}{{R}_{sh}}-{I}_{L}\\\:x=\left[{I}_{ph},{I}_{R1},{I}_{R2},{R}_{s},{R}_{sh},{\alpha\:}_{1},{\alpha\:}_{2}\:\:\right]\end{array}\right.$$

For the TDD, $\:{f}_{L}\left({I}_{L},{V}_{L},x\right)$ and $\:\left(x\right)$ can be expressed as:

17 $$\:\left\{\begin{array}{c}{f}_{L}\left({I}_{L},{V}_{L},x\right)={I}_{ph}-{I}_{R1}\left[exp\left(\frac{e\left({V}_{L}+{I}_{L}{R}_{s}\right)}{{\alpha\:}_{1}KT}\right)-1\right]-{I}_{R2}\left[exp\left(\frac{e\left({V}_{L}+{I}_{L}{R}_{s}\right)}{{\alpha\:}_{2}KT}\right)-1\right]-\\\:{I}_{R3}\left[exp\left(\frac{e\left({V}_{L}+{I}_{L}{R}_{s}\right)}{{\alpha\:}_{3}KT}\right)-1\right]-\frac{{V}_{L}+{I}_{L}{R}_{s}}{{R}_{sh}}-{I}_{L}\\\:x=\left[{I}_{ph},{I}_{R1},{I}_{R2},{I}_{R3},\:{R}_{s},{R}_{sh},{\alpha\:}_{1},{\alpha\:}_{2},{\alpha\:}_{3}\:\:\right]\end{array}\right.$$

## Electric EEL foraging optimization algorithm

As previously stated, optimization algorithms play an important role in tackling complicated, nonlinear issues across various fields, including data science and engineering. MH algorithms have garnered significant attention in recent years for their effectiveness in exploring complex search spaces while avoiding local optima. Among these, the electric eel foraging optimization (EEFO) algorithm^45^ stands out as an innovative approach inspired by the foraging behavior of electric eels. Electric eels use electrical discharges to identify and grab prey in murky waters, balancing exploration with exploitation. The EEFO algorithm simulates this behavior, utilizing simulated electric fields to traverse the search space and identify optimal solutions. The electric discharge method allows the algorithm to study new locations (exploration) while also fine-tuning solutions in promising places (exploitation). EEFO’s capacity to balance exploitation and exploration enables it to be particularly successful at addressing nonlinear optimization problems with many solutions and restrictions. Its electric field-inspired approach effectively guides the search process, preventing early convergence and avoiding local optima—common challenges in other algorithms. The exploitation and exploration stages of EEFO are modeled after the social predation behaviors of electric eels, including interaction, rest, migration, and hunting.

### Interacting

EEFO uses a collaborative method inspired by eels’ social predation, with each eel representing a potential solution. At each stage, the best solution serves as the target, whereas eels communicate with randomly picked partners, modifying their positions depending on the distance between them and the population’s center or a random point in the search space. This method includes “churn,” which is a random movement in several directions to improve exploration. The churn is mathematically expressed as:

18 $$\begin{gathered} C = n_{1} \times B \hfill \\ n_{1} \sim N\left( {0,1} \right) \hfill \\ B = \left[ {b_{1} ,b_{2} , \ldots b_{k} , \ldots b_{d} } \right] \hfill \\ \end{gathered}$$

In this context, the function *b(k)* equals 1 when *k* equals *g*, and *b(k)* equals 0 for all other values of *k*. The value of *g* is determined by creating a random permutation of the numbers from 1 to d. The interactive behaviour is characterized as:

19 $$\:\left\{\begin{array}{c}\left\{\begin{array}{c}{v}_{i}\left(t+1\right)={x}_{j}\left(t\right)+C\times\:\left(\stackrel{-}{x}\left(t\right)-{x}_{i}\left(t\right)\right),\:\:\:\:\:{p}_{1}>0.5\\\:{v}_{i}\left(t+1\right)={x}_{j}\left(t\right)+C\times\:\left({x}_{r}\left(t\right)-{x}_{i}\left(t\right)\right),\:\:\:\:\:{p}_{1}\le\:0.5\end{array}\:\:\:\:\:\:\:\:\:\:{f}_{it}\left({x}_{j}\left(t\right)\right)<{f}_{it}\left({x}_{i}\left(t\right)\right)\right.\\\:\left\{\begin{array}{c}{v}_{i}\left(t+1\right)={x}_{i}\left(t\right)+C\times\:\left(\stackrel{-}{x}\left(t\right)-{x}_{j}\left(t\right)\right),\:\:\:\:\:{p}_{2}>0.5\\\:{v}_{i}\left(t+1\right)={x}_{i}\left(t\right)+C\times\:\left({x}_{r}\left(t\right)-{x}_{j}\left(t\right)\right),\:\:\:\:\:{p}_{2}\le\:0.5\end{array}\right.\:\:\:\:\:\:\:\:\:{f}_{it}\left({x}_{j}\left(t\right)\right)\ge\:{f}_{it}\left({x}_{i}\left(t\right)\right)\end{array}\right.$$

where the variables *p*_*1*_ and *p*_*2*_ denote random numbers selected from the interval between 0 and 1, the fitness of the proposed position of the *ith* electric eel is defined as $\:{f}_{it}\left(x\left(t\right)\right)$, *x*_*r*_ is determined as the product of the lower bound (Low) and the sum of a random number (*r*) multiplied by the difference between the upper bound (Up) and the lower bound, and *x*_*j*_ is the position of an eel randomly selected from the current population, with *j ≠ i*. Additionally, the formula for $\:\stackrel{-}{x}\left(t\right)\:$ is expressed as follows.

20 $$\:\stackrel{-}{x}\left(t\right)=\frac{1}{n}\sum\:_{i=1}^{n}{x}_{i}\left(t\right)$$

where n denotes the population size, as given in Eq. (20). The movement behaviour of electric eels enables them to traverse various regions within the search space, significantly enhancing the exploration capabilities of the EEFO algorithm.

### Resting

The EEFO algorithm establishes a specified resting area before eels exhibit resting behavior. To improve the search process, this location is where one dimension of the eel’s position vector crosses the main diagonal of the search space. The resting region is calculated by normalizing the search area and the position of the eel, which ranges from 0 to 1, then projecting a random dimension onto the main diagonal to obtain the center of the resting zone. Equation (21) defines this resting pattern.

21 $$\:{v}_{i}\left(t+1\right)={R}_{i}\left(t+1\right)+{n}_{2}\times\:\left({R}_{i}\left(t+1\right)-rond\left(rand\right)\times\:{x}_{i}\left(t\right)\right)$$

where *n*_*2*_ is a continuation of a normal distribution with a mean of 0 and standard deviation of 1 and *R*_*i*_ denotes the resting posture.

### Hunting

Electric eels employ a hunting strategy that extends beyond mere swarming upon locating their target. Instead, they cooperate by forming a large circular configuration to surround the target. Eels employ moderate electric organ discharges to communicate and collaborate. As eels interact, the electrified circle size decreases. Finally, the eels guide the group of fish from deeper waters to shallower areas, making them easier to catch. In accordance with this behavior, the electrified circle functions as the designated hunting area. At this stage, the target makes calculated movements within the hunting area, often changing locations out of fear. The hunting behavior exhibited by eels, characterized by their curling motion, can be described via Eq. (22) as follows:

22 $$\:{v}_{i}\left(t+1\right)={H}_{prey}\left(t+1\right)+\eta\:\times\:\left({H}_{prey}\left(t+1\right)-rond\left(rand\right)\times\:{x}_{i}\left(t\right)\right)$$

where *η* denotes the curling factor, which was introduced in^53^, and *H*_*prey*_ reflects the current location of the prey in relation to its previous position within the hunting area.

### Migrating

Upon detecting the scent of prey, electric eels naturally migrate from their resting areas to their hunting grounds. To quantify this migratory behavior, the following equation is used.

23 $$\:{v}_{i}\left(t+1\right)={{-r}_{5}\times\:R}_{i}\left(t+1\right)+{r}_{6}\times\:\left({H}_{r}\left(t+1\right)-L\times\:{H}_{r}\left(t+1\right)-{x}_{i}\left(t\right)\right)$$

In this case, *H*_*r*_ refers to any location within the hunting region, and the arbitrary numbers from (0, 1) denote *r*_*5*_ and *r*_*6*_, respectively. The Levy flight function (*L*) is used in the exploitation stage of EEFO to avoid local optima.

The MEEFO algorithm builds on the advantages of EEFO by introducing improvements that enhance its search efficiency, leading to better solution accuracy and faster convergence. These modifications make the algorithm more capable of addressing complex optimization problems with increased robustness and precision.

## Modified electric eel foraging optimization algorithm

The suggested MEEFO is based on boosting the searching capability of the traditional EEFO via three strategies. It should be highlighted here that the main feature of meta heuristic optimization is that each optimizer should has its unique exploitation and exploration mechanisms and for boosting the searching abilities these optimizers, it can be accomplished via improving the exploitation and exploration process. At the end of this context, the exploitation phase of the proposed optimizer has been improved by integration fractional-order calculus (FOC) while the exploration phase has been boosted via the fitness distance balance (FDB) strategy, fractional-order calculus (FOC), and quasi opposition-based learning (QOBL).

### The fractional-order calculus strategy

FC calculus is a novel tool and a hot topic that has been integrated with optimization methods. The FO calculus is based on the use of the memory properties of FO calculus for boosting the exploitation core of the optimization techniques. Grünwald–Letnikov is one of the most commonly used methods for modelling the FO calculus for a discrete segment ($\:{X}_{i}^{t}$), which can be presented as follows^54^:

24 $$\:{D}^{\sigma\:}\left[{X}_{i}^{t}\right]=\frac{1}{{T}^{\sigma\:}}\sum\:_{n=0}^{r}\:\frac{(-1{)}^{n}{\Gamma\:}(\sigma\:+1){X}_{i}^{(t-nT)}}{{\Gamma\:}(n+1){\Gamma\:}(\sigma\:-n+1)}$$

where $\:{\Gamma\:}$ is the gamma function. $\:r$ is the number of memories, and $\:T$ refers to the sampling period. In this paper, $\:r$ is selected to be 4. Hence, the FO calculus that can be applied to the optimization problem can be represented as follows:

25 $$\begin{aligned} x_{i}^{{new}} = & \frac{1}{{1!}}\sigma X_{i}^{{\left( t \right)}} + \frac{1}{{2!}}\sigma \left( {1 - \sigma } \right)X_{i}^{{\left( {t - 1} \right)}} + \frac{1}{{3!}}\sigma \left( {1 - \sigma } \right)\left( {2 - \sigma } \right)X_{i}^{{\left( {t - 2} \right)}} \\ & \quad + \frac{1}{{4!}}\sigma \left( {1 - \sigma } \right)\left( {2 - \sigma } \right)\left( {3 - \sigma } \right)X_{i}^{{\left( {t - 3} \right)}} \\ & \quad \; + \left. {\frac{1}{{5!}}\sigma \left( {1 - \sigma } \right)\left( {2 - \sigma } \right)\left( {3 - \sigma } \right)\left( {4 - \sigma } \right)X_{i}^{{\left( {t - 4} \right)}} } \right| \\ \end{aligned}$$

$\:\sigma\:$ is a value from 0.1 to 1.

### The fitness distance balance strategy

In this strategy, the positions of the eels are adjusted according to the FDB strategy, in which eel locations are updated depending upon the values of the corresponding objective functions of eel locations and the distance between each eel and the best location. The following steps describe the FDB strategy:

*Step 1* Construct the location matrix of the eel locations and the corresponding objective function as follows.

26 $$X \equiv \left[ {\begin{array}{*{20}c} {x_{{11}} } & \cdots & {x_{{1,D}} } \\ \vdots & \ddots & \vdots \\ {x_{{N1}} } & \cdots & {x_{{N,D}} } \\ \end{array} } \right]$$

27 $$\:Obj=\left[{Obj}_{1},{Obj}_{2},\ldots\:,{Obj}_{N}\right]$$

*Step 2* Find the spacing balance between the positions of the *i-*th eel and their prey, and construct the distance matrix as follows.

28 $$\:{DM}_{i}=\sqrt{\begin{array}{c}{\left({x}_{i,1}-{x}_{prey,1}\right)}^{2}+{\left({x}_{i,2}-{x}_{prey,2}\right)}^{2}+\cdots\:\\\:+{\left({x}_{i,D}-{x}_{prey,D}\right)}^{2}\end{array}}$$

29 $$\:DM=\left[{DM}_{1},{DM}_{2},\cdots\:,{DM}_{n}\right]$$

*Step 3* Find the score of FDB, which can be calculated as follows.

30 $$\:{Scor}_{i}=\gamma\:\times\:\left(1-\text{n}\text{o}\text{r}\text{m}{obj}_{i}\right)+(1-\gamma\:)\times\:\text{n}\text{o}\text{r}\text{m}{DM}_{i}$$

In which

31 $$\:\text{n}\text{o}\text{r}\text{m}{DM}_{i}=\frac{{DM}_{i}-{DM}_{min}}{{DM}_{max}-{DM}_{min}}$$

32 $$\:\text{n}\text{o}\text{r}\text{m}{obj}_{i}=\frac{{obj}_{i}-{obj}_{min}}{{obj}_{max}-{obj}_{min}}$$

where $\:max$ and $\:min$ are the maximum and minimum values, respectively. $\:\gamma\:$ is selected to be 0.5^55^.

### The Quasiopposition-based learning strategy

The idea of OBL for boosting the performance of optimization techniques was presented by Tizhoosh^3^. OBL depends on assigning opposite locations to populations that have a high probability of better approximation than the current populations, which can be described as follows:

33 $$\:O{X}_{i,j}={Up}_{j}+{Lp}_{j}-{X}_{i,j};\:\forall\:\:i\in\:N;\:\forall\:\:j\in\:D$$

The quasiopposite population means finding the reflect point that also has a probability of being closer to the best population, which can be assigned as follows^56,57^:

34 $$x_{{i,j}}^{{new}} = \left\{ {\begin{array}{*{20}l} {\frac{{Up_{j} + Lp_{j} }}{2} + \left( {OX_{{i,j}} - \frac{{Up_{j} + Lp_{j} }}{2}} \right) \times rand} \hfill & {If~~\left( {X_{{i,j}} < \frac{{Up_{j} + Lp_{j} }}{2}~} \right)} \hfill \\ {\frac{{Up_{j} + Lp_{j} }}{2} + \left( {\frac{{Up_{j} + Lp_{j} }}{2} - OX_{{i,j}} } \right) \times rand} \hfill & {else} \hfill \\ \end{array} } \right.$$

Finally, the new updated solutions are accepted or rejected on the basis of their corresponding objective functions via greedy selection as follows:

35 $$x_{i} ^{{t + 1}} = \left\{ {\begin{array}{*{20}l} {x_{i}^{{new}} } \hfill & {{\text{if}}\;{\text{Obj}}\left( {x_{i}^{{new}} } \right) < Obj\left( {x_{i} ^{t} } \right)} \hfill \\ {x_{i} ^{t} } \hfill & {{\text{otherwise~}}} \hfill \\ \end{array} } \right.$$

**Fig. 4:**
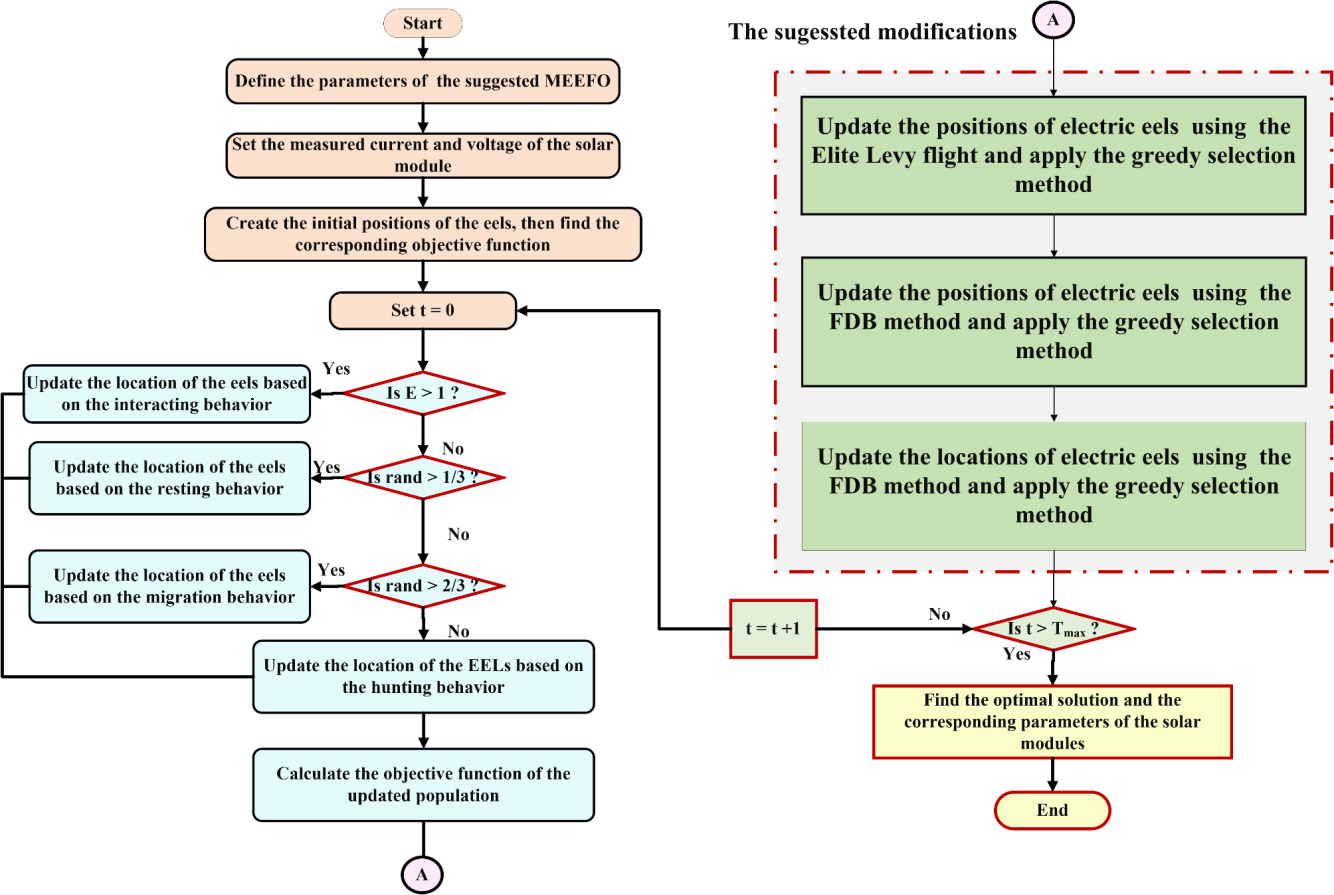
Flowchart of the MEEFO for PV module parameter identification.

Figure 4 lists the steps of MEEFO for parameter estimation of solar PV modules.

### Code availability

Readers can access the proposed modified algorithm in the supplementary files provided with the published paper.

## Simulation validation and analysis

The proposed MEEFO is utilized for assigning the solar PV module’s parameters and for assessing the performance of the MEEFO. Both standard functions and CEC-2019 functions have been examined. The simulations were conducted via MATLAB R2022a, and the results captured by MEEFO were compared with those of other techniques, such as BDO^48^, ZOA^49^, AVOA^50^ and HHO^51^, the sand cat search optimizer (SCSO)^52^ and the traditional EEFO.

### Validation of MEEFO on the standard benchmark

The MEEFO performance is validated on the standard functions and the CEC-2019 functions. The standard functions comprise a set of single-modal functions (F1–F7), multimodal functions (F8–F13), and fixed-dimensional functions (F14–F23). The details of the benchmark function are given in^56,57^. The parameters of the algorithms under study are displayed in Table 1. The population size, maximum number of iterations, and number of trial runs were selected to be 30, 500, and 25, respectively. The importance of this act is having compromise between best solution and run times.

#### Statistical validation

The statistical outcomes of the suggested technique with the alternative competing techniques are listed in Table 2, including the best, worst, mean, and standard deviation (St.d.) values. The values highlighted in bold in Table 2 represent the best results. As depicted in Table 2, the findings obtained by the MEEFO for the single-modal functions (F1–F7) are better than those of the other competitor techniques, which demonstrates that the MEEFO has high exploitation ability. The performance of MEEFO is the same as that of EEFO for F6. For the multimodal functions (F8–F13) that have multiple local optima and one global optimum, the statistical results of these benchmarks demonstrate that the suggested optimization method has a high exploration core. Notably, the performance of MEEFO is identical to that of the other reported techniques for most functions, such as F9-F12-F13-F16-F17-F18. Similarly, the fixed-dimensional multimodal functions F14 to F23 have fewer local optima than the other functions (F8–F13). All these functions (F1 to F23) are defined and extracted from^58^. Thus, the fixed dimension can be utilized to evaluate the equilibrium between exploration ability and exploitation ability. According to the obtained results, MEEFO has a high ability to reach equilibrium between exploitation and exploration performance, in which MEEFO has a similar or optimal solution in relation to the other methods.

#### Convergence characteristics

**Fig. 5:**
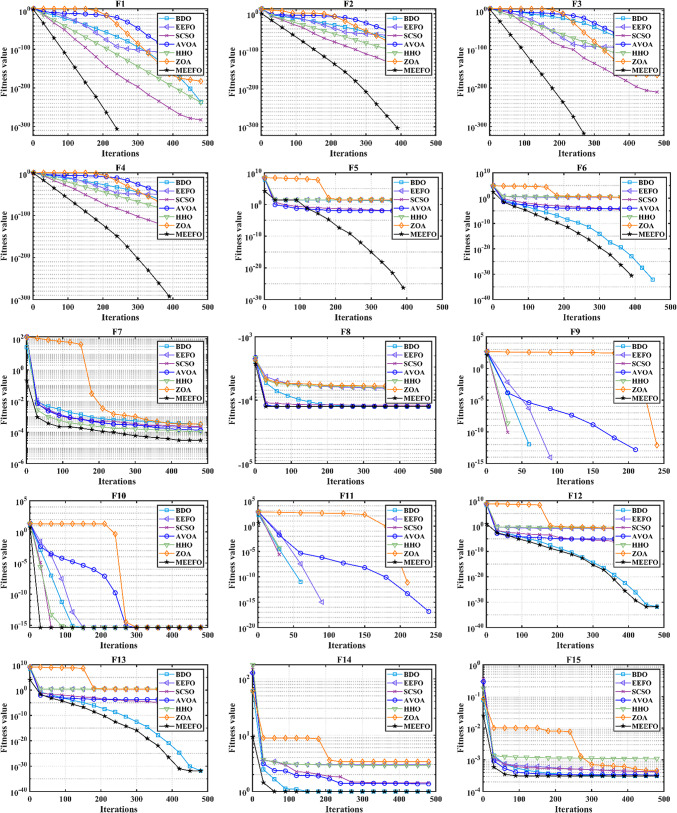

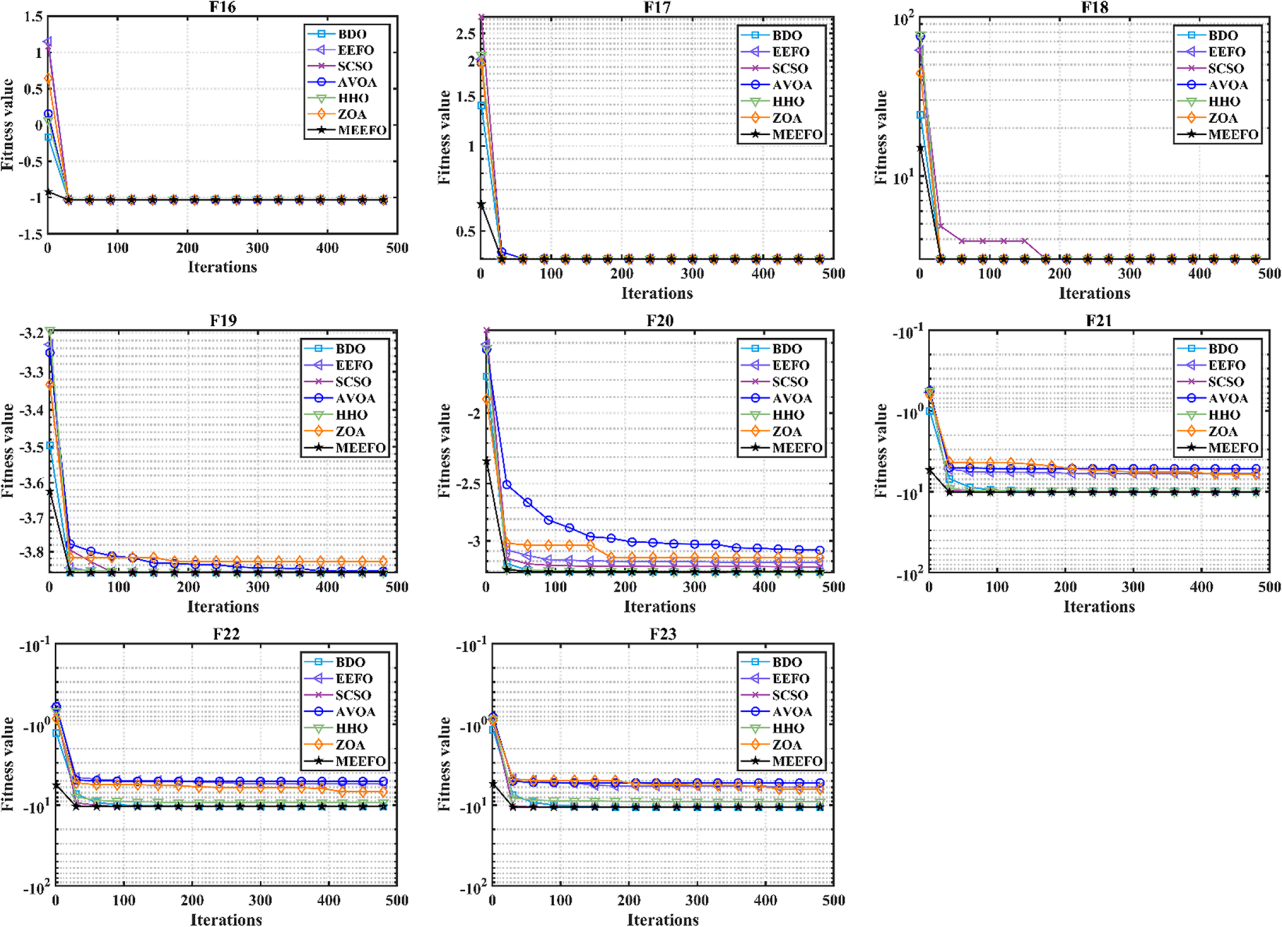
The convergence plots of MEEFO against BDO, ZOA, SCSO, HHO and the traditional EEFO for the standard functions.

The convergence properties of MEEFO and comparative optimization methods such as BDO, the ZOA, SCSO, HHO, and traditional EEFO for the standard benchmark are shown in Fig. 5. It is clear that the suggested MMEFO converges rapidly for F1–F7 (the unimodal functions), which verifies that the exploitation performance of the MEEFO is better than that of the reported optimization techniques. Additionally, the MEEFO converges rapidly for F8–F13 (the multimodal functions). Similarly, for the fixed-dimensional functions (F14–F23), the suggested MMEFO converges before iteration number 100.

#### Boxplot validation

**Fig. 6:**
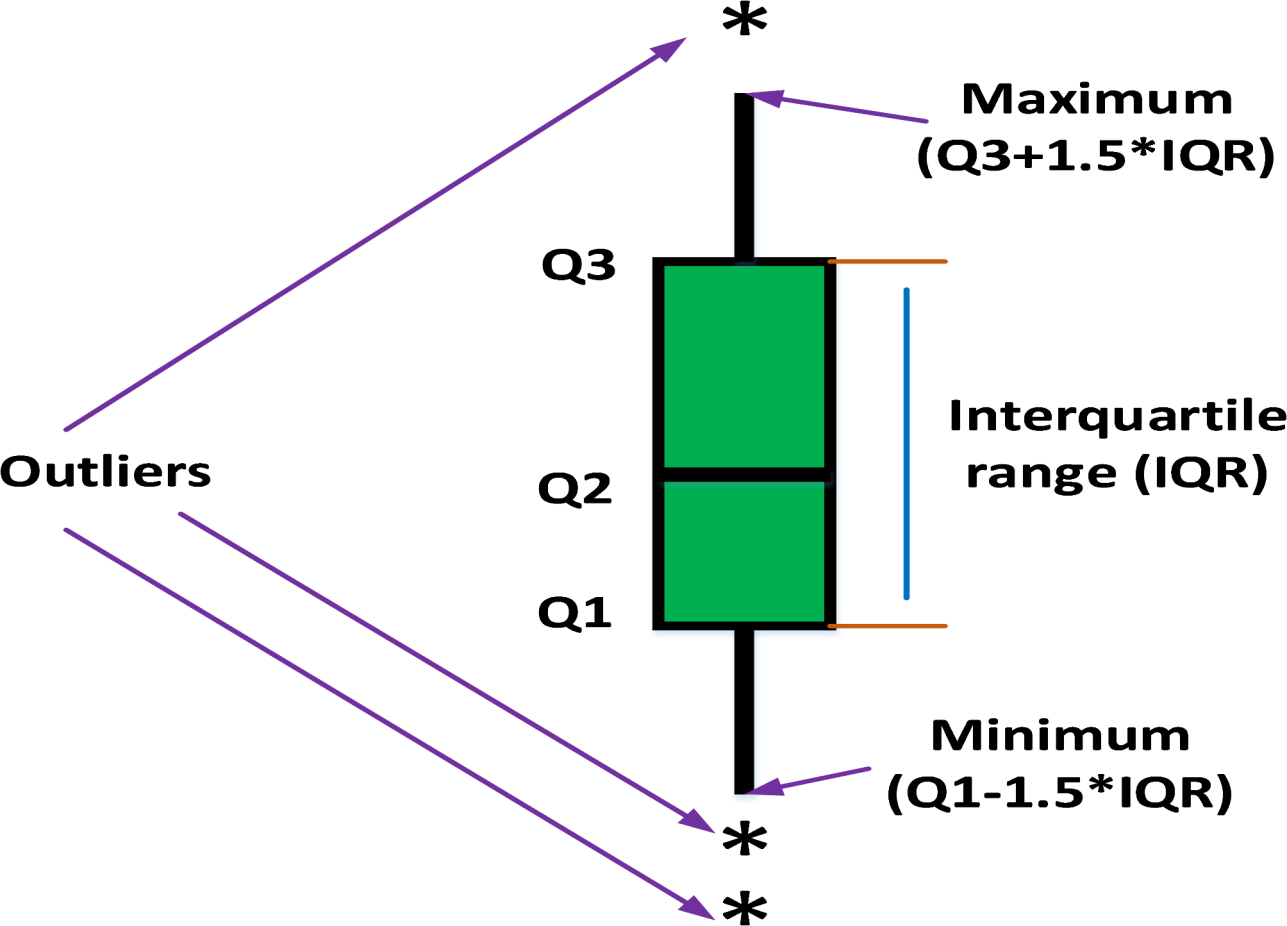
The boxplot representation.

**Fig. 7:**
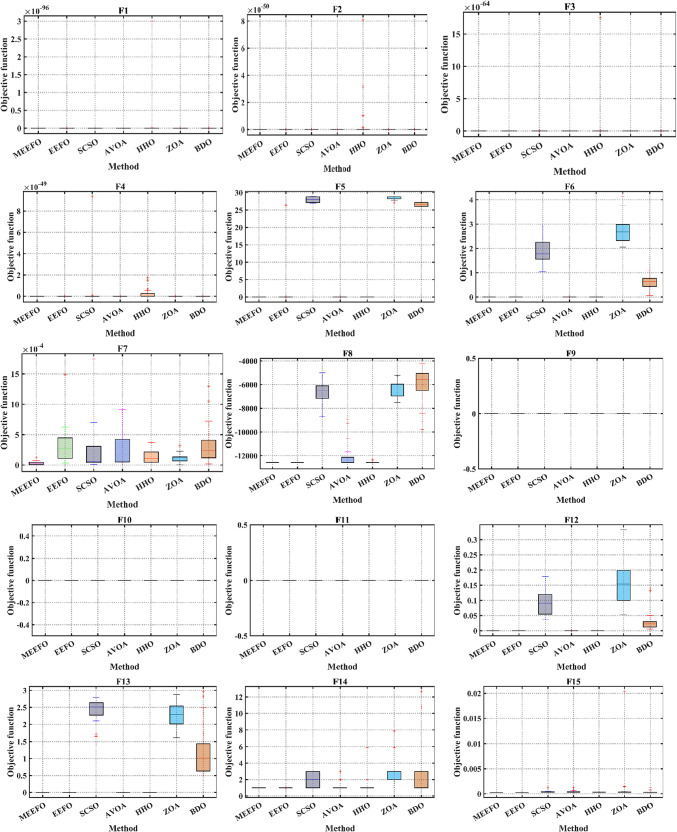

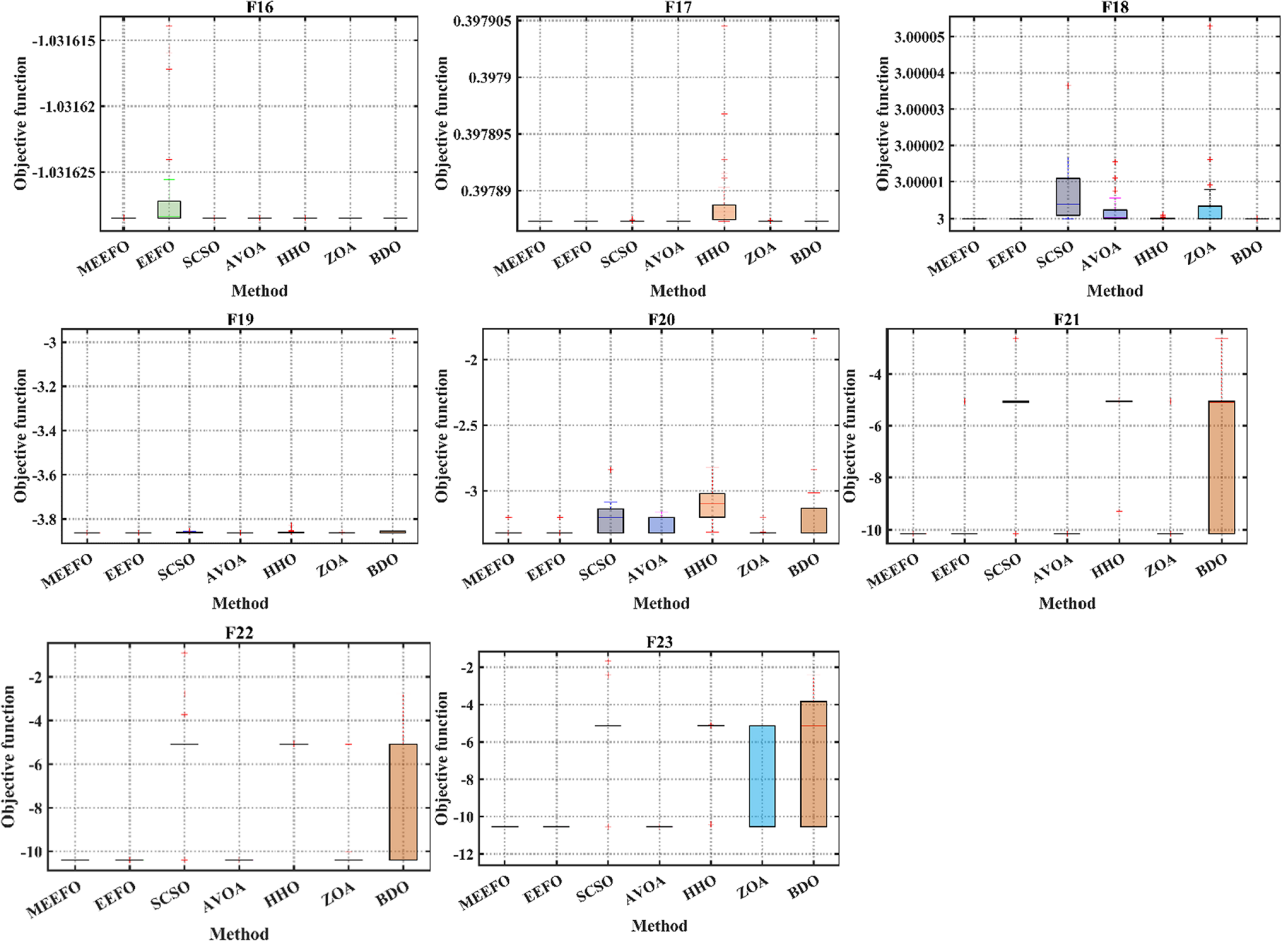
The boxplots of MEEFO against BDO, ZOA, SCSO, HHO and the traditional EEFO for the standard functions.

Further assessing the performance and searching ability of boxplots is a common method for illustrating the distribution of the capture results^6^. The schematic of the boxplot is shown in Fig. 6, where Q2 refers to the median value of the results, while the 25th and 75th percentiles are represented by Q1 and Q3, respectively. Furthermore, outliers represent values of or less than the minimum and greater than the maximum values. Figure 7 shows the boxplot of the classical benchmark functions that were obtained from the results of MEEFO against BDO, ZOA, SCSO, HHO, and the traditional EEFO. The boxplot of MEEFO is clearly the best for most of the functions in which the minimum median value and the narrowest boxplot are obtained via the application of MEEFO, which proves the superior performance of MEEFO.

#### Nonparametric test validation

Two nonparametric tests are used to compare the captured results with those of other optimization techniques to verify the performance of MEEFO. The Wilcoxon signed rank method is the first method that has been widely applied for comparing the results of two optimizers and determining whether there is a difference between these results or not using the $\:p-value$ index^59^. If $\:p-value>0.5$, this verifies that there is no notable difference between the obtained results, but if $\:p-value<0.5$, this verifies that there is a notable difference between the yielded results. The $\:p-value$ values between MMEFO and BDO, the ZOA, SCSO, HHO, and the traditional EEFO are listed in the 7th column of Table 2. Table 2 clearly shows that $\:p-value$ is less than 0.05 for most optimizers, indicating that there are meaningful differences between the obtained results. However, for some cases, $\:p-value$ is greater than 0.05, such as F14 against EEFO and F16 against ZOA. Consequently, there is no large difference in the results for these cases. Notably, the NAN in Table 2 refers to the fact that the results between the optimizers cannot be compared because the data are similar.

**Fig. 8:**
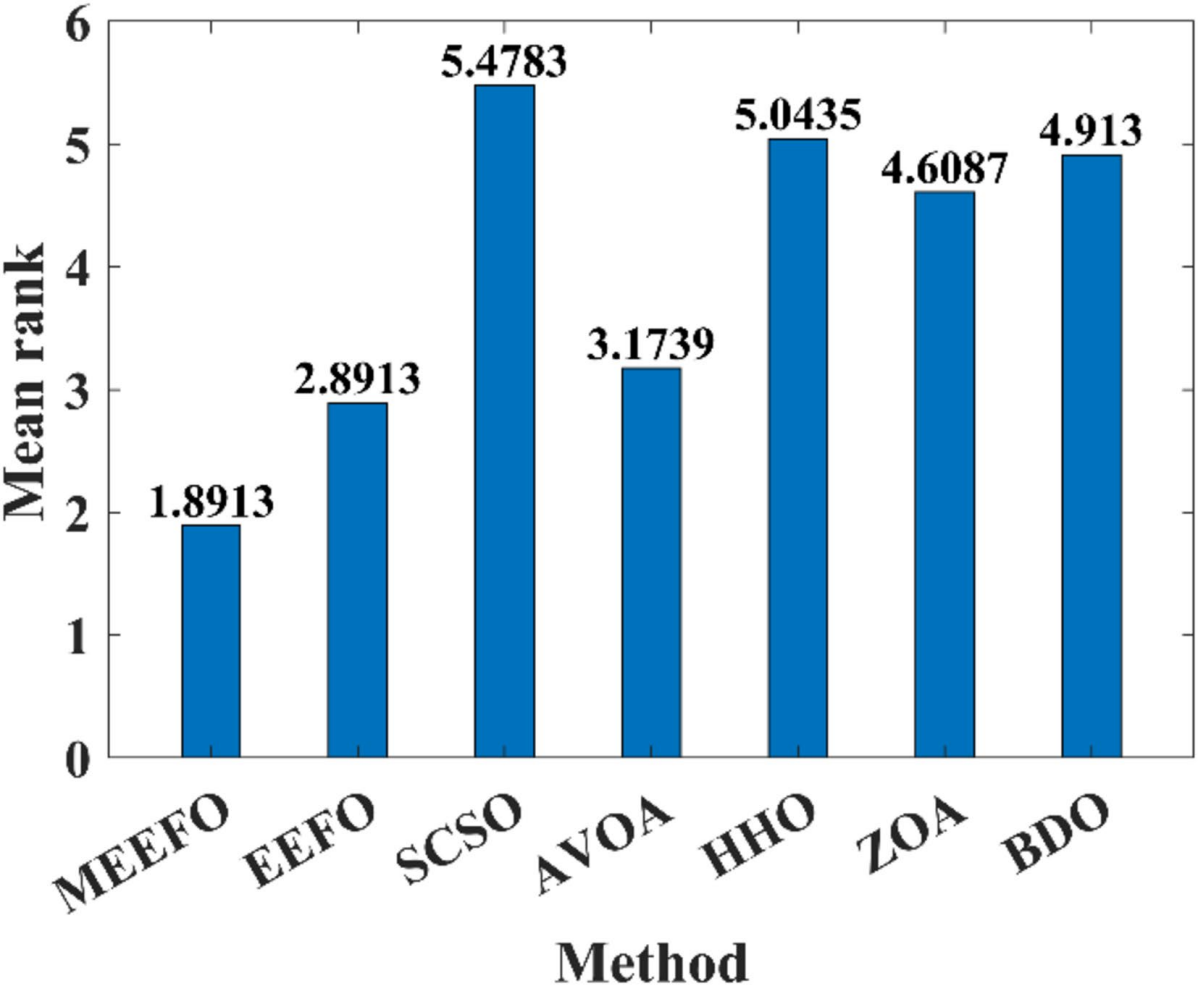
The ranking of the optimizers using the Freiman test.

The second nonparametric metric is Friedman’s rank test (FRT), which is used to evaluate the performance of the MEEFO compared with that of the other techniques; this metric is based on the ranking of the optimizer using their mean values and the $\:p-value$ index that can be calculated for the FRT^60^, in which the lowest mean value indicates the best optimization method. Figure 8 shows the rankings of MMEFO and BDO, the ZOA, SCSO, HHO, and EEFO. The MEEFO method clearly has the lowest mean, and the best performance compared with those of the other comparative methods.

**Table 1:** The parameters of the algorithms studied.

Algorithm	Parameters
BDO^48^	$\:k=\lambda\:=1.5,\:b=0.3,\:S=0.5.$
SCSO^52^	$\:rg=\left[\text{2,0}\right],\:R=\left[-\:2rg,\:2rg\right]$
AVOA^50^	$\:w=2.5,P1=0.2,\:P2=2.5,\:\:P3=2.5,\:L1=0.8,L2=0.2$
GWO^61^	$\:a=\left[\text{2,0}\right]$
HHO^51^	$\:{E}_{0}=[-\text{1,1}],\:\:\beta\:=1.5$
ZOA^49^	$\:R=0.01$
EEFO	*b* = 1.5
MEEFO	*b* = 1.5

**Table 2:** The statistical comparative results of MEEFO against BDO, ZOA, SCSO, HHO, and the traditional EEFO for standard functions.

	Algorithms	min	mean	worst	St.d	$p-value$
F1	MEEFO	**0**	**0**	**0**	**0**	–
	EEFO	9.88082E−284	8.91563E−265	2.66947E−263	0	1.21178E−12
	SCSO	3.25469E−126	3.31552E−113	9.90008E−112	1.80723E−112	1.21178E−12
	AVOA	**0**	2.75279E−291	8.25836E−290	0	1.30556E−07
	HHO	8.61251E−115	1.00623E−97	2.99989E−96	5.47589E−97	1.21178E−12
	ZOA	2.34153E−261	4.78256E−250	6.39988E−249	0	1.21178E−12
	BDO	4.78786E−198	2.70094E−185	6.90650E−184	0	1.21178E−12
F2	MEEFO	**0**	**0**	**0**	**0**	–
	EEFO	1.67041E−147	2.68826E−134	7.99294E−133	1.45889E−133	1.21178E−12
	SCSO	2.92145E−65	4.32319E−60	1.20266E−58	2.19068E−59	1.21178E−12
	AVOA	3.99745E−176	2.87343E−151	8.53334E−150	1.55747E−150	1.21178E−12
	HHO	2.41635E−60	4.18470E−51	8.07155E−50	1.56421E−50	1.21178E−12
	ZOA	7.14980E−136	3.76598E−131	7.60417E−130	1.39508E−130	1.21178E−12
	BDO	2.10375E−104	6.28709E−96	1.82905E−94	3.33634E−95	1.21178E−12
F3	MEEFO	**0**	**0**	**0**	**0**	–
	EEFO	6.79322E−229	3.13282E−189	9.37177E−188	0	1.21178E−12
	SCSO	4.37885E−111	1.22386E−96	3.59952E−95	6.56815E−96	1.21178E−12
	AVOA	1.72968E−283	5.36089E−212	1.60742E−210	0	1.21178E−12
	HHO	6.97244E−109	5.83636E−65	1.75091E−63	3.19670E−64	1.21178E−12
	ZOA	1.70810E−173	4.40393E−156	7.99108E−155	1.61922E−155	1.21178E−12
	BDO	2.89457E−186	1.22705E−168	2.17228E−167	0	1.21178E−12
F4	MEEFO	**0**	**0**	**0**	**0**	–
	EEFO	4.36604E−137	3.37733E−129	7.26219E−128	1.33221E−128	1.21178E−12
	SCSO	1.68904E−56	3.20793E−50	9.36955E−49	1.70910E−49	1.21178E−12
	AVOA	1.60175E−176	3.58094E−144	8.27191E−143	1.56076E−143	1.21178E−12
	HHO	1.37666E−55	2.61170E−50	1.72477E−49	5.06231E−50	1.21178E−12
	ZOA	1.10320E−119	1.02049E−114	2.46268E−113	4.47074E−114	1.21178E−12
	BDO	1.87268E−96	8.68189E−89	1.26873E−87	3.08608E−88	1.21178E−12
F5	MEEFO	0	0	0	0	–
	EEFO	0	8.81623E−01	2.64183E+01	4.82310	1.94574E−09
	SCSO	2.69742E+01	2.79762E+01	2.88907E+01	7.48848E−01	1.21178E−12
	AVOA	3.84550E−07	5.36345E−05	2.89740E−04	5.61842E−05	1.21178E−12
	HHO	1.01909E−04	1.02425E−02	3.18350E−02	9.41188E−03	1.21178E−12
	ZOA	2.71307E+01	2.84895E+01	2.88277E+01	4.20610E−01	1.21178E−12
	BDO	2.53186E+01	2.67270E+01	2.87608E+01	9.54374E−01	1.21178E−12
F6	MEEFO	**0**	**0**	**0**	**0**	–
	EEFO	**0**	**0**	**0**	**0**	NAN
	SCSO	1.03339	1.87694	2.98389	4.49744E−01	1.21178E−12
	AVOA	1.55292E−08	4.97783E−07	1.24681E−06	2.27465E−07	1.21178E−12
	HHO	1.48368E−06	7.42605E−05	2.37838E−04	7.59995E−05	1.21178E−12
	ZOA	2.05584	2.77486	4.13557	5.35829E−01	1.21178E−12
	BDO	6.64838E−02	6.24705E−01	1.15984	2.68355E−01	1.21178E−12
F7	MEEFO	9.15928E−07	**2.94735E**−**05**	**1.20454E**−**04**	**2.73721E**−**05**	–
	EEFO	3.35423E−05	3.07732E−04	1.48523E−03	2.82356E−04	5.96731E−09
	SCSO	9.49088E−06	2.10220E−04	1.74271E−03	3.40069E−04	5.97056E−05
	AVOA	**4.87799E**−**07**	2.38969E−04	9.13798E−04	2.27055E−04	1.4918E−06
	HHO	7.51555E−06	1.34648E−04	3.74343E−04	1.13781E−04	1.01877E−05
	ZOA	5.75290E−06	1.06432E−04	3.17095E−04	6.31274E−05	1.25408E−07
	BDO	1.76423E−05	3.20286E−04	1.29637E−03	2.97336E−04	6.72195E−10
F8	MEEFO	**− 1.25695E+04**	**− 1.25695E+04**	**− 1.25695E+04**	**1.85009E−12**	–
	EEFO	− **1.25695E+04**	− **1.25695E+04**	− **1.25695E+04**	**1.85009E−12**	NAN
	SCSO	− 8.71985E+03	− 6.64882E+03	− 5.00928E+03	8.77106E+02	1.21178E−12
	AVOA	− 1.25695E+04	− 1.19871E+04	− 8.66097E+03	1.12621E+03	1.21178E−12
	HHO	− 1.25695E+04	− 1.25614E+04	− 1.23473E+04	4.05077E+01	1.21178E−12
	ZOA	− 7.50004E+03	− 6.53013E+03	− 5.20868E+03	6.50697E+02	1.21178E−12
	BDO	− 9.78563E+03	− 6.00394E+03	− 4.20418E+03	1.38359E+03	1.21178E−12
F9	MEEFO	0	0	0	0	–
	EEFO	0	0	0	0	NAN
	SCSO	0	0	0	0	NAN
	AVOA	0	0	0	0	NAN
	HHO	0	0	0	0	NAN
	ZOA	0	0	0	0	NAN
	BDO	0	0	0	0	NAN
F10	MEEFO	4.441E−16	4.441E−16	4.441E−16	0	–
	EEFO	4.441E−16	4.441E−16	4.441E−16	0	NAN
	SCSO	4.441E−16	4.441E−16	4.441E−16	0	NAN
	AVOA	4.441E−16	4.441E−16	4.441E−16	0	NAN
	HHO	4.441E−16	4.441E−16	4.441E−16	0	NAN
	ZOA	4.441E−16	4.441E−16	4.441E−16	0	NAN
	BDO	4.441E−16	4.441E−16	4.441E−16	0	NAN
F11	MEEFO	0	0	0	0	–
	EEFO	0	0	0	0	NAN
	SCSO	0	0	0	0	NAN
	AVOA	0	0	0	0	NAN
	HHO	0	0	0	0	NAN
	ZOA	0	0	0	0	NAN
	BDO	0	0	0	0	NAN
F12	MEEFO	**1.57054E**−**32**	**1.57054E**−**32**	**1.57054E**−**32**	**5.56740E**−**48**	–
	EEFO	**1.57054E**−**32**	**1.57054E**−**32**	**1.57054E**−**32**	**5.56740E**−**48**	NAN
	SCSO	3.66453E−02	9.00984E−02	1.78879E−01	4.06461E−02	1.21178E−12
	AVOA	2.05146E−09	2.64340E−08	7.33065E−08	1.84423E−08	1.21178E−12
	HHO	7.06662E−09	8.45856E−06	5.36436E−05	1.32553E−05	1.21178E−12
	ZOA	5.24551E−02	1.59783E−01	3.31047E−01	6.69370E−02	1.21178E−12
	BDO	5.20898E−03	2.79202E−02	1.39058E−01	3.10921E−02	1.21178E−12
F13	MEEFO	**1.34978E**−**32**	**1.34978E**−**32**	**1.34978E**−**32**	**5.56740E**−**48**	–
	EEFO	**1.34978E**−**32**	**1.34978E**−**32**	**1.34978E**−**32**	**5.56740E**−**48**	NAN
	SCSO	1.42117	2.41182	2.79202	3.34202E−01	1.2117E−12
	AVOA	1.51956E−09	3.29579E−08	1.28400E−07	3.23602E−08	1.2117E−12
	HHO	1.99622E−07	1.87225E−04	1.33353E−03	2.89174E−04	1.2117E−12
	ZOA	1.61564	2.28279	2.87604	3.21679E−01	1.2117E−12
	BDO	4.80779E−01	1.19039	2.96791	7.43736E−01	1.2117E−12
F14	MEEFO	9.9800E−01	**9.9800E**−**01**	**9.9800E**−**01**	**0**	–
	EEFO	9.9800E−01	**9.9800E**−**01**	**9.9800E**−**01**	7.14170E−17	8.14042E−02
	SCSO	9.98004E−01	3.02753	1.07632E+01	3.22260	1.21178E−12
	AVOA	9.9800E−01	1.42796	2.98211	8.10751E−01	1.79328E−09
	HHO	9.98004E−01	1.29490	5.92885	9.39939E−01	1.21178E−12
	ZOA	9.98004E−01	2.90750	7.87399	2.11264	1.20586E−12
	BDO	9.98004E−01	3.37857	1.26705E+01	3.99024	5.61606E−09
F15	MEEFO	**3.07486E**−**04**	**3.07486E**−**04**	**3.07486E**−**04**	**1.20799E**−**19**	–
	EEFO	**3.07486E−04**	3.07560E**−**04	3.09716E**−**04	4.07168E**−**07	1.67381E**−**10
	SCSO	3.07487E**−**04	4.12663E**−**04	1.22318E**−**03	2.34585E**−**04	2.87183E**−**11
	AVOA	3.07566E**−**04	4.21496E**−**04	1.22317E**−**03	1.94032E**−**04	2.87183E**−**11
	HHO	3.07507E**−**04	3.32363E**−**04	3.73000E**−**04	1.84716E**−**05	2.87183E**−**11
	ZOA	3.07503E**−**04	1.08270E**−**03	2.03633E**−**02	3.65242E**−**03	2.87183E**−**11
	BDO	**3.07486E−04**	4.47148E**−**04	1.22317E**−**03	2.99871E**−**04	2.87183E**−**11
F16	MEEFO	− 1.03163	− 1.03163	− 1.03163	7.38241E−09	–
	EEFO	− 1.03163	− 1.03163	− 1.03161	4.37062E−06	1.24807E−04
	SCSO	− 1.03163	− 1.03163	− 1.03163	5.45797E−10	1.97665E−05
	AVOA	− 1.03163	− 1.03163	− 1.03163	**4.441E−16**	1.38525E−01
	HHO	− 1.03163	− 1.03163	− 1.03163	2.34295E−09	2.75037E−02
	ZOA	− 1.03163	− 1.03163	− 1.03163	7.39010E−10	4.00914E−01
	BDO	− 1.03163	− 1.03163	− 1.03163	5.63848E−16	6.95263E−03
F17	MEEFO	3.97887E−01	3.97887E−01	3.97887E−01	0	–
	EEFO	3.97887E−01	3.97887E−01	3.97887E−01	0	NAN
	SCSO	3.97887E−01	3.97887E−01	3.97888E−01	3.41613E−08	1.21178E−12
	AVOA	3.97887E−01	3.97887E−01	3.97887E−01	0	NAN
	HHO	3.97887E−01	3.97889E−01	3.97905E−01	3.51383E−06	1.21178E−12
	ZOA	3.97887E−01	3.97887E−01	3.97887E−01	1.50678E−08	1.94446E−09
	BDO	3.97887E−01	3.97887E−01	3.97887E−01	0	NAN
F18	MEEFO	3	3	3	**1.16624E**−**15**	–
	EEFO	3	3	3	1.79729E−15	0.009853469
	SCSO	3	3.00001	3.00004	7.58285E−06	2.25645E−11
	AVOA	3	3	3.00002	4.38341E−06	2.25645E−11
	HHO	3	3	3	2.61079E−07	2.25645E−11
	ZOA	3	3	3.00005	9.95061E−06	7.47892E−09
	BDO	3	3	3	3.20768E−15	0.037649739
F19	MEEFO	−3.86278	−3.86278	−3.86278	2.69625E−15	−
	EEFO	− 3.86278	− 3.86278	− 3.86278	**2.65431E−15**	0.169400748
	SCSO	− 3.86278	− 3.86113	− 3.85490	2.96588E− 03	1.72025E−12
	AVOA	− 3.86278	− 3.86278	− 3.86278	3.40664E−12	2.18113E−12
	HHO	− 3.86278	− 3.85992	− 3.82963	6.29728E−03	1.72025E−12
	ZOA	− 3.86278	− 3.86225	− 3.86032	6.12490E−04	1.72025E−12
	BDO	− 3.86278	− 3.82904	− 2.98440	1.59573E−01	4.24714E−09
F20	MEEFO	− 3.32200	− 3.31011	− 3.20310	3.62777E−02	–
	EEFO	− 3.32200	− 3.30614	− 3.20310	4.11068E−02	0.334156166
	SCSO	− 3.32199	− 3.21040	− 2.84042	1.16902E−01	2.41783E−10
	AVOA	− 3.32200	− 3.25686	− 3.15981	6.24522E−02	3.26035E−10
	HHO	− 3.31638	− 3.10284	− 2.81879	1.25079E−01	3.84844E−11
	ZOA	− 3.32200	− 3.31734	− 3.20146	**2.19835E**−**02**	2.24832E−08
	BDO	− 3.32200	− 3.16166	− 1.84092	2.76968E−01	2.80957E−06
F21	MEEFO	− 1.0153E+01	− **1.0153E+01**	− **1.0153E+01**	**6.8481E**−**15**	–
	EEFO	− 1.0153E+01	− 9.81333	− 5.05520	1.29340	9.71718E−05
	SCSO	− 1.0153E+01	− 5.91616	− 2.63046	2.23960	7.57407E−12
	AVOA	− 1.0153E+01	− **1.0153E+01**	− **1.0153E+01**	2.33601E−13	7.53676E−12
	HHO	− 9.31387	− 5.19397	− 5.02941	7.78144E−01	7.57407E−12
	ZOA	− 1.01532E+01	− 9.98309	− 5.05519	9.30731E−01	7.57407E−12
	BDO	− 1.01532E+01	− 6.11333	− 2.63047	2.85036	9.32712E−11
F22	MEEFO	− 1.0403E+01	− **1.0403E+01**	− **1.0403E+01**	8.07992E−16	–
	EEFO	− 1.0403E+01	− **1.0403E+01**	− **1.0403E+01**	**4.66494E**−**16**	0.0300607
	SCSO	− 1.04029E+01	− 5.44343	− 9.11615E−01	2.17838	6.31878E−12
	AVOA	− 1.0403E+01	− **1.0403E+01**	− **1.0403E+01**	8.38934E−13	6.27819E−12
	HHO	− 5.08759	− 5.08295	− 5.06956	5.50927E−03	6.31878E−12
	ZOA	− 1.04029E+01	− 9.50371	− 5.08765	2.00997	6.31878E−12
	BDO	− 1.04029E+01	− 6.83451	− 2.76590	3.03747	3.38708E−07
F23	MEEFO	− 1.0536E+01	− **1.0536E+01**	− **1.0536E+01**	**8.07992E−16**	–
	EEFO	− 1.0536E+01	− **1.0536E+01**	− **1.0536E+01**	1.54719E−15	0.000674799
	SCSO	− 1.0534E+01	− 6.00477	− 1.67654	2.43308	6.31878E−12
	AVOA	− 1.0536E+01	− **1.05364E+01**	− **1.05364E+01**	3.05735E−13	8.51206E−12
	HHO	− 1.0412E+01	− 5.30006	− 5.08969	9.65600E−01	6.31878E−12
	ZOA	− 1.0536E+01	− 9.09420	− 5.12835	2.43232	6.31878E−12
	BDO	− 1.0536E+01	− 6.33041	− 2.42734	3.12100	1.12812E−09

### Validation of the effect of MEEFO on the CEC-2019 function

**Fig. 9:**
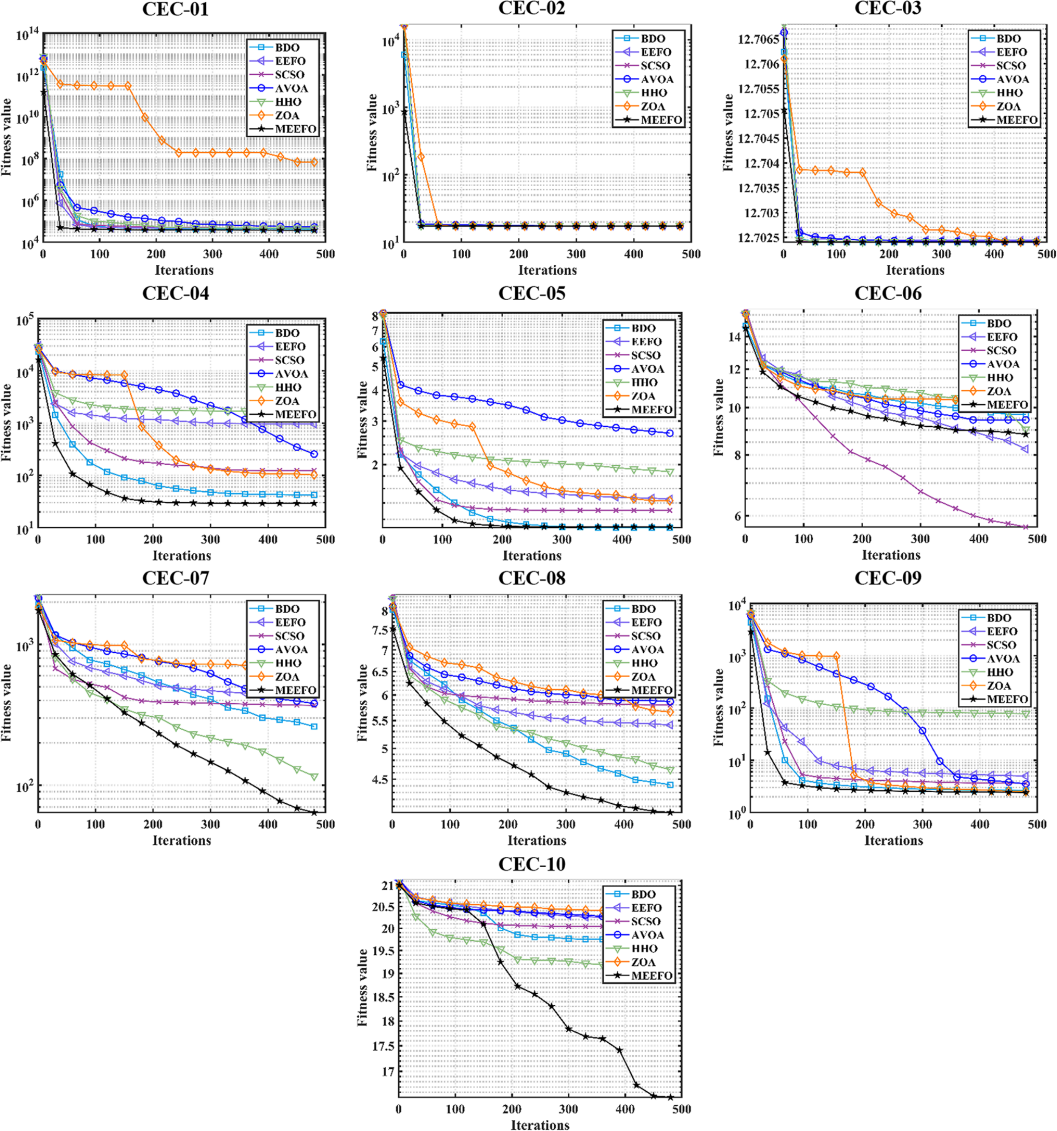
The convergence plots of MEEFO against BDO, ZOA, SCSO, HHO, and the traditional EEFO for CEC-2019 functions.

**Fig. 10:**
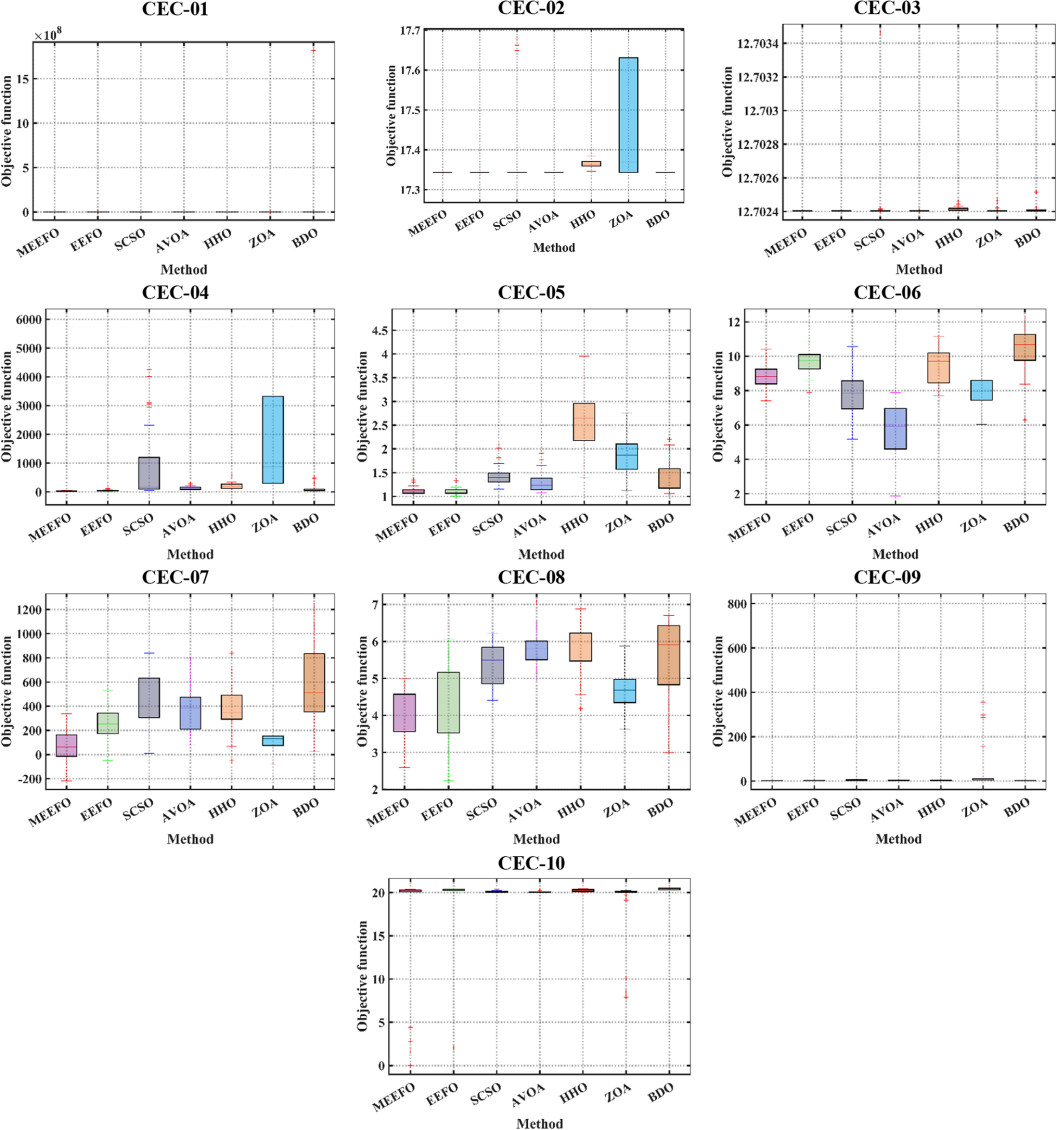
The boxplots of CEC-2019 function of the applied optimizers.

To further assess the performance of the proposed technique, the CEC-2019 test suite, which is considered one of the newest methods, consists of 10 multimodal functions, and the descriptions of these functions are given in^62,63^. The statistical results obtained for MEEFO, BDO, ZOA, SCSO, HHO, and EEFO for CEC-2019 are given in Table 3, in which the optimal results are highlighted in bold. As per the reported results in Table 3, the MEEFO is superior in terms of the best, mean, and worst values for most functions. However, the performance is less than that of EEFO for CEC-05 and less than that of AVOA for CEC-06. The convergence characteristics of the reported techniques for CEC-2019 are shown in Fig. 9. According to Fig. 9, the MEEFO converges to the optimal solution before the 200th iteration for CEC01 to CEC-05. However, the performance of MEEFO is worse than that of SCSO for CEC06 in terms of convergence characteristics. Finally, the boxplot for CEC 2019 is displayed in Fig. 10. The results of the proposed method MEEFO compared to the results of some published algorithms are displayed in Table A1 [Appendix].

**Table 3:** The statistical comparative results of MEEFO against BDO, ZOA, SCSO, HHO, and the traditional EEFO for the CEC-2019 test suit.

	Algorithms	Min	Mean	Worst	St.d	*p* value
CEC-01	MEEFO	**33075.2542**	**36776.7056**	**40568.266**	**1.8074E+03**	–
	EEFO	38758.0103	42045.2055	52488.629	2.7711E+03	2.1544E−10
	SCSO	40191.8859	45755.9017	65615.357	5.3798E+03	3.6897E−11
	AVOA	40777.7458	46980.3230	56362.180	4.2395E+03	3.0198E−11
	HHO	40832.2126	52738.8196	68526.424	6.7437E+03	3.0198E−11
	ZOA	38763.7542	46385.5470	122857.046	1.4713E+04	6.0657E−11
	BDO	36991.8033	60710347.9118	1.8202E+09	3.3231E+08	1.5964E−07
CEC-02	MEEFO	**17.342857**	**17.342857**	**17.342857**	**7.1360E**−**15**	–
	EEFO	**17.342857**	**17.342857**	**17.342857**	3.3373E−11	7.30901E−12
	SCSO	17.342934	17.375126	17.677837	9.7713E−02	1.72025E−12
	AVOA	**17.342857**	**17.342857**	17.342858	1.9980E−07	1.72025E−12
	HHO	17.346393	17.364433	17.383418	9.1437E−03	1.72025E−12
	ZOA	17.342908	17.472468	17.683559	1.4346E−01	1.72025E−12
	BDO	**17.342857**	**17.342857**	**17.342857**	8.9489E−15	2.17315E−05
CEC-03	MEEFO	12.702404	**12.702404**	**12.702404**	**3.6134E**−**15**	–
	EEFO	12.702404	**12.702404**	**12.702404**	**3.6134E**−**15**	NAN
	SCSO	12.702404	12.702441	12.703463	1.9316E−04	1.2117E−12
	AVOA	12.702404	12.702404	**12.702404**	8.0975E−11	5.5947E−09
	HHO	12.702405	12.702415	12.702457	1.2171E−05	1.2117E−12
	ZOA	12.702404	12.702409	12.702465	1.4551E−05	1.2117E−12
	BDO	12.702404	12.702410	12.702516	2.0503E−05	1.29742E−07
CEC-04	MEEFO	**2.9849**	**29.3512**	**58.7022**	**1.3538E+01**	–
	EEFO	8.9568	42.4882	120.3904	2.5466E+01	2.51013E−02
	SCSO	48.7944	958.0441	4256.4559	1.3095E+03	4.07716E−11
	AVOA	40.7943	124.2348	269.6238	5.3649E+01	1.20567E−10
	HHO	64.4456	206.4057	573.7037	1.1023E+02	3.01986E−11
	ZOA	37.8609	1695.2275	6063.3391	1.7521E+03	6.06576E−11
	BDO	23.8790	101.1331	480.5720	1.1365E+02	9.83289E−08
CEC-05	MEEFO	1.0123	1.1199	1.345	7.5528E−02	–
	EEFO	**1.0074**	**1.1108**	**1.330**	**6.3874E**−**02**	7.61828E−01
	SCSO	1.1504	1.4327	2.088	2.2818E−01	8.10136E−10
	AVOA	1.0688	1.3052	1.909	2.1289E−01	3.83494E−06
	HHO	1.4006	2.6476	4.771	7.3358E−01	3.01986E−11
	ZOA	1.1220	1.8638	2.751	4.1058E−01	3.47420E−10
	BDO	1.0616	1.4180	2.201	3.2697E−01	1.38525E−06
CEC-06	MEEFO	7.4022	8.8030	10.429	7.0478E−01	–
	EEFO	7.8858	9.6955	10.848	**6.8062E**−**01**	1.09069E−05
	SCSO	5.1846	7.7350	10.574	1.3118	3.77040E−04
	AVOA	**1.8787**	**5.6639**	**7.890**	1.5610	6.06576E−11
	HHO	7.6941	9.4397	11.147	1.0063	1.17107E−02
	ZOA	6.0374	7.9649	9.803	8.9657E−01	3.77040E−04
	BDO	6.2978	10.3406	12.257	1.2460	4.80107E−07
CEC-07	MEEFO	**− 216.8623**	**61.7885**	**337.929**	**1.2468E+02**	–
	EEFO	**− **49.6213	232.8089	529.461	1.4322E+02	2.13273E−05
	SCSO	10.8509	422.5551	838.587	2.1104E+02	1.10234E−08
	AVOA	80.5001	369.0671	795.224	1.6894E+02	6.51827E−09
	HHO	− 50.0915	373.1823	838.418	1.8785E+02	1.31110E−08
	ZOA	− 78.4126	111.5378	229.328	6.8655E+01	8.49997E−02
	BDO	27.3917	588.7128	1260.133	3.3768E+02	5.46175E−09
CEC-08	MEEFO	2.5955	**3.9969**	**5.000**	5.8828E−01	–
	EEFO	**2.2321**	4.3507	6.073	1.0552	1.11987E−01
	SCSO	4.4103	5.3983	6.215	5.5278E−01	1.28704E−09
	AVOA	4.9330	5.7967	7.060	**4.6226E**−**01**	3.68973E−11
	HHO	4.1802	5.8428	6.876	6.1144E−01	2.37147E−10
	ZOA	3.6235	4.6234	5.881	5.3206E−01	1.68132E−04
	BDO	2.9940	5.5744	6.705	1.0586	5.18568E−07
CEC-09	MEEFO	**2.3452**	**2.4229**	**2.573**	**5.2545E**−**02**	–
	EEFO	2.4116	2.6423	3.377	2.1558E−01	6.01039E−08
	SCSO	2.9261	4.8919	8.530	1.4225	3.01986E−11
	AVOA	2.4748	3.6117	5.299	6.8255E−01	4.50432E−11
	HHO	2.4408	3.3557	4.789	5.1484E−01	5.49405E−11
	ZOA	3.3586	77.7609	806.222	1.7366E+02	3.01986E−11
	BDO	2.3767	2.5204	2.881	1.2685E−01	5.26404E−04
CEC-10	MEEFO	**2.50E**−**07**	**16.5021**	**20.400**	7.6776	–
	EEFO	2.0134	19.7218	20.496	3.3455	1.68132E−04
	SCSO	20.0019	20.1383	20.413	1.1261E−01	4.05950E−02
	AVOA	19.9943	20.0373	20.252	6.7315E−02	3.56384E−04
	HHO	19.9899	20.2408	20.479	**1.2389E**−**01**	3.55472E−01
	ZOA	7.9087	18.9441	20.224	3.4517	1.00353E−03
	BDO	20.0539	20.3928	20.646	1.6058E−01	1.86817E−05

### Application of MEEFO for parameter identification of PV modules

In this section, the proposed HGTO-BWO is applied to experimental data to address the critical issue of determining the optimal parameters for the equivalent circuit of PV cells/panels. The topic is crucial since it calls for the creation of a trustworthy, realistic model of a PV system. This facilitates numerous studies being conducted in the built circuit using the suggested methods. Under typical circumstances, the characteristics have been estimated for R.T.C. France, PVM752, STM6-40/36, PWP-201, and STP6-120/36. Additionally, the SQ-150 double diode variants were built at different temperatures and sun irradiances. The upper and lower bounds of the design variables are displayed in Table 4. To ensure an equitable comparison between the proposed MEEFO and the other techniques, 30 runs were carried out for each optimizer under consideration, a maximum of 500 iterations were set, and the size of the population was set at 30.

**Table 4:** Minimum and maximum design variable limits for certain PV cell/model combinations.

Parameters	R.T.C		PVW 752		STP6-120/36		PWP-201		STM6-40/36		SQ-150	
	L_b_	U_b_	L_b_	U_b_	L_b_	U_b_	L_b_	U_b_	L_b_	U_b_	L_b_	U_b_
${\alpha }_{1}$ *, * ${\alpha }_{2}$ *,* ${\alpha }_{3}$	1	2	1	2	1	50	1	50	1	60	1	2
*R*_*s*_	0	0.5	0	0.8	0	0.36	0	2	0	0.36	0	2
*R*_*sh*_	0	100	0	1000	0	1500	0	2000	0	1000	0	1000
*I*_*d1*_, *I*_*d2*_, *I*_*d3*_	0	1E−6	0	1E−6	0	50E−6	0	50E−6	0	50E−6	0	10E−6
*I*_*pv*_	0	1	0	0.5	0	8	0	2	0	2	0	10

## PV results

### PV cells

**Fig. 11:**
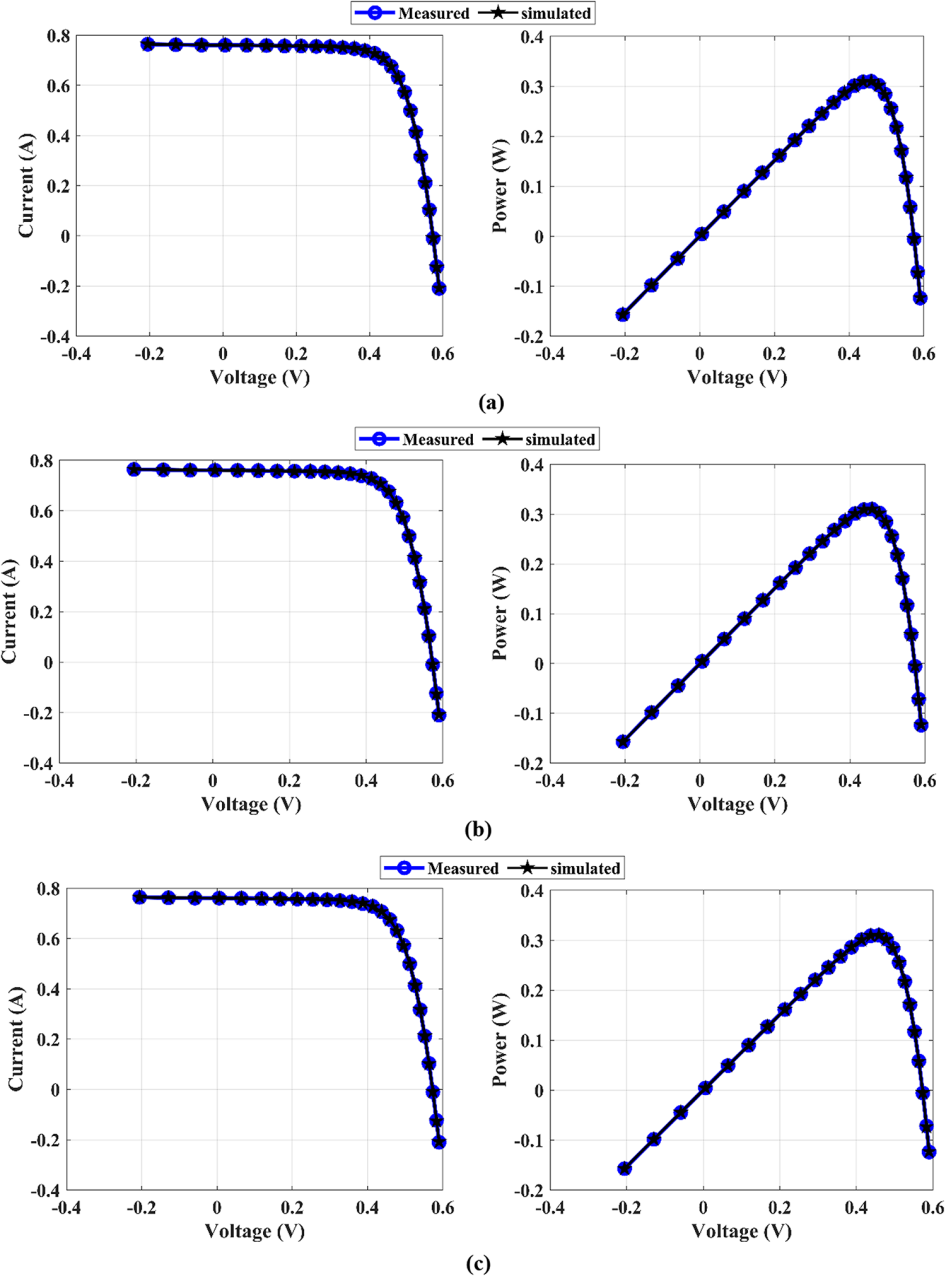
The *I*–*V* and *P*–*V* curves for RTC France: (**a**) SDM, (**b**) DDM, and (**c**) TDM

**Fig. 12:**
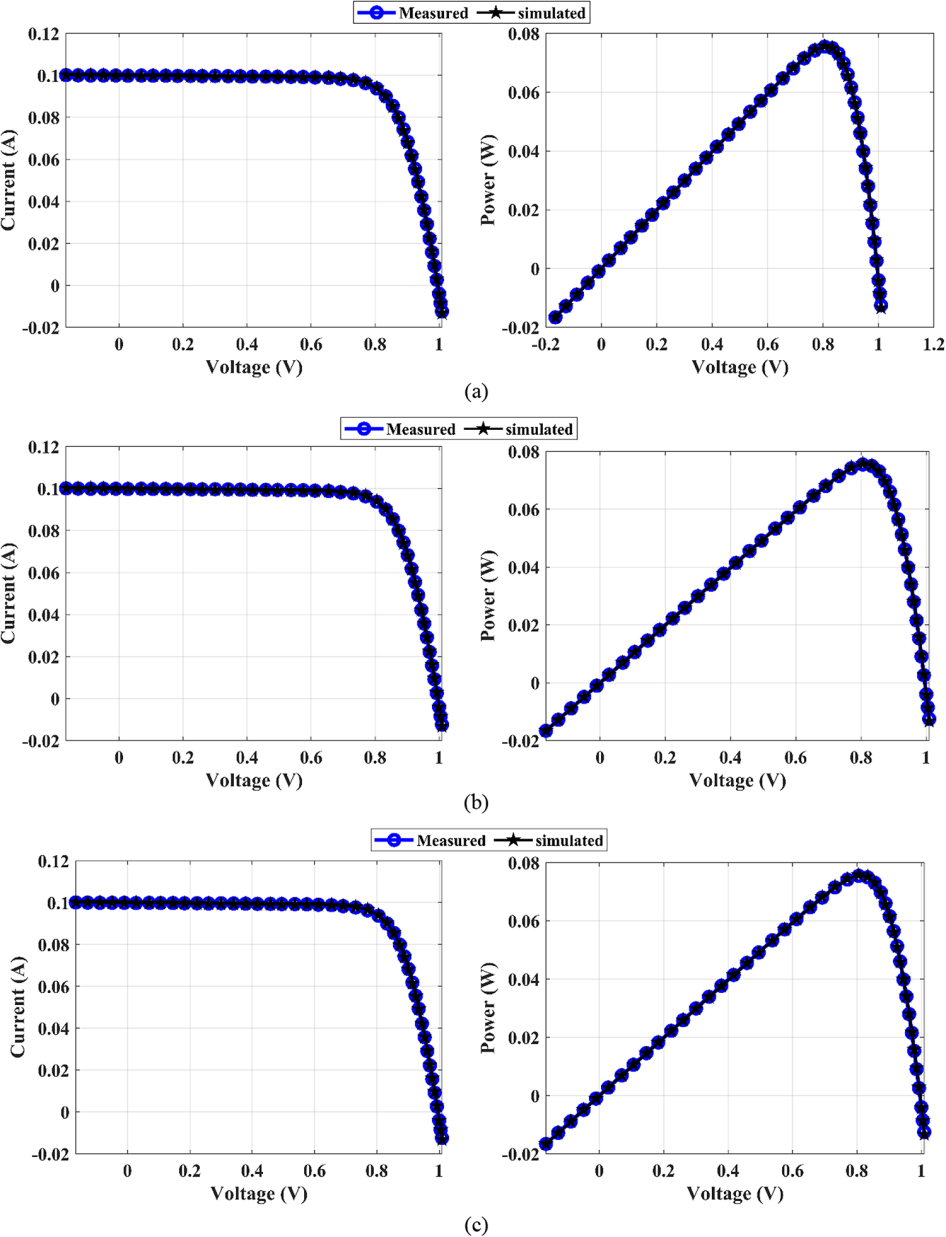
*I*–*V* and *P*–*V* curves for PVW752 cell: (**a**) SDM, (**b**) DDM, and (**c**) TDM

**Fig. 13:**
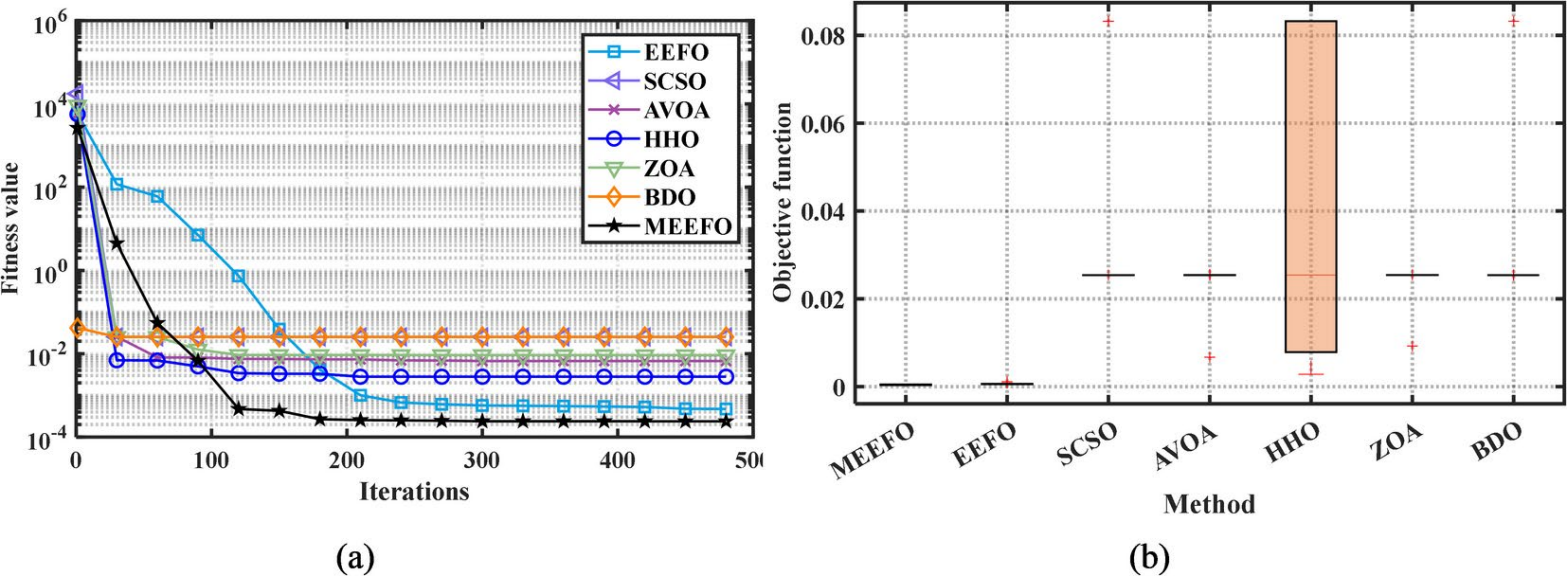
The convergence plots and boxplots for TDM-PVW752.

The MEEFO algorithm is used to determine the parameters of the PV models; the SDM, DDM, and TDM are used for the R.T.C. France PV and PVM752 at certain temperatures and solar irradiance. The unit cell characteristics and the measured data for the I‒V curves are illustrated in^64,65^. The RMSE values and the optimum parameters of the SDM via the MEEFO optimization technique are provided in Table 5. This table shows a comparison between the MEEFO algorithm and the other algorithms, while the MEEFO algorithm achieves the lowest value of the RMSE, with values of 9.861273E-04 for the RTC-France and 2.886069E-04 for the PVW 752 solar cell module. The worst values are those of the AVOA algorithm, with RMSE values of 9.371689E-03 and 9.701927E-03 in RTC-France and PVW 752, respectively. For the DDM, the optimal parameters extracted throughout the MEEFO in comparison with the other introduced agathisms for the RTC France and PVW 752-unit cell are organized in Table 6. The MEEFO algorithm achieves the lowest RMES value of 9.83945E-04, placing it in the top rank for the RTC France and 2.36081E-04 for the PVW 752, whereas the second one belongs to the EEFO, which gives a 9.89241E-04 RMSE value for the RTC France and 4.04340E-04 for the PVW 752-unit cell. The values of the TDM’s optimal parameters are introduced in Table 7. This table reveals that the lowest value of RMSE, 9.83820E-04, belongs to MEEFO, whereas the worst value comes from HHO, with a value of 3.26458E-03 for the RTC France unit cell. For the PVW 752-unit cell, the lowest RMSE value is 2.39004E-04, which comes from the MEEFO. On the other hand, the RMSE worst value is introduced via the ZOA method, with a value of 9.25658E-03. The measured and simulated (I–V) and (P–V) curves of the SDM, DDM, and TDM for RTC France and PVW752 PV cells, which are distinguished throughout the suggested MEEFO algorithm, are displayed in Figs. 11 and 12. The convergence plots and boxplots for TDM-PVW752 displayed in Fig. 13.

**Table 5:** The optimal SDM parameters for the RTC France and PVW752 unit cells.

	Algorithms	α	R_s_	R_sh_	I_d1_	I_pv_	RMSE
RTC	MEEFO	1.481748	0.036354	53.8539	3.2484E−07	0.76078	**9.861273E**−**04**
	EEFO	1.560523	0.033059	73.2621	6.8151E−07	0.76075	1.793210E−03
	SCSO	1.645987	0.024105	9.7014	1.3363E−06	0.78052	1.535841E−02
	AVOA	1.997037	0.013794	99.9999	1.4245E−05	0.76605	9.371689E−03
	HHO	1.647653	0.031307	78.7203	1.4255E−06	0.76329	4.668810E−03
	ZOA	1.160837	0.039718	5.1962	5.1175E−09	0.79105	2.465254E−02
	BDO	10	00	1.1489	0.0000	0.83682	2.228614E−01
PVW 752	MEEFO	1.714228	0.626223	788.9193	1.5041E−11	0.09999	**2.886069E**−**04**
	EEFO	1.835559	0.582387	979.1746	6.7166E−11	0.10001	4.483524E−04
	SCSO	1.395948	00	34.8712	6.6297E−14	0.10492	1.036582E−02
	AVOA	1.492140	0.000246	37.2335	4.0855E−13	0.10455	9.701927E−03
	HHO	1.755981	0.711841	889.7974	2.5214E−11	0.10037	2.199206E−03
	ZOA	1.124168	0.001689	14.5879	0.0000	0.11377	2.540252E−02
	BDO	20	00	14.5886	0.0000	0.11376	2.539958E−02

**Table 6:** The optimal DDM parameters for the RTC France and PVW752 unit cells.

Type	Algorithms	α_1_	α_2_	R_s_	R_sh_	I_o1_	I_o2_	I_pv_	RMSE
RTC	MEEFO	1.45625	1.87117	0.03663	54.771	2.373E−07	3.863E−07	0.76078	**9.83945E**−**04**
	EEFO	1.43096	1.76451	0.03693	53.616	1.682E−07	4.393E−07	0.76083	9.89241E−04
	SCSO	1.47144	1.84755	0.03657	92.489	2.794E−07	3.042E−07	0.75944	1.43849E−03
	AVOA	1.88520	1.47185	0.03685	58.300	2.915E−09	2.945E−07	0.76033	1.06254E−03
	HHO	1.44894	1.87434	0.03730	70.913	2.244E−07	2.737E−07	0.75914	1.39721E−03
	ZOA	1.43434	1.71407	0.03630	54.555	1.585E−07	4.937E−07	0.76120	1.09010E−03
	BDO	2	1.47424	0.03660	49.639	0.00	3.012E−07	0.76090	1.00646E−03
PVW 752	MEEFO	1.79764	1.59501	0.64991	635.639	1.075E−11	2.076E−12	0.10004	**2.36081E**−**04**
	EEFO	1.56607	1.86250	0.59726	960.344	3.842E−13	7.144E−11	0.09996	4.04340E−04
	SCSO	1.80923	1.06606	0.53510	126.844	4.742E−11	0.00	0.10252	1.73083E−03
	AVOA	1.54769	1.86570	0	24.864	0.00	6.942E−11	0.11438	9.89138E−03
	HHO	2	2	0.14104	201.116	1.919E−10	1.817E−10	0.09874	5.72329E−03
	ZOA	1.00246	1.04421	0.00009	14.589	0.00	0.00	0.11376	2.53997E−02
	BDO	2	2	0	14.589	0.00	0.00	0.11376	2.53996E−02

**Table 7:** The optimal parameters of TDM for the RTC France and PVW752 unit cells.

Type	Algo.	α_1_	α_2_	α_3_	R_s_	R_sh_	I_d1_	I_d2_	I_d3_	I_pv_	RMSE
RTC	MEEFO	1.9186	1.9229	1.4637	0.0366	54.593	3.943E−08	2.955E−07	2.614E−07	0.76078	**9.83820E**−**04**
	EEFO	1.4979	1.8304	1.7046	0.0357	60.702	3.778E−07	3.859E−08	1.603E−09	0.76090	1.07419E−03
	SCSO	1.4136	1.7345	1.9807	0.0362	42.670	1.255E−07	5.859E−07	1.201E−08	0.76183	1.40391E−03
	AVOA	1.7173	1.4575	1.8683	0.0362	91.822	2.291E−07	2.220E−07	2.558E−07	0.75954	1.34665E−03
	HHO	1.5931	1.8304	1.8829	0.0313	84.198	8.224E−07	3.012E−08	6.244E−07	0.76027	3.26458E−03
	ZOA	1.4378	1.4533	1.7375	0.0376	60.729	1.000E−07	1.097E−07	1.973E−07	0.76042	1.26474E−03
	BDO	2.0000	2.0000	1.4379	0.0370	55.578	1.000E−06	0	1.936E−07	0.76080	9.83746E−04
PVW 752	MEEFO	1.6045	1.7464	1.9258	0.6470	699.080	2.055E−12	8.139E−12	2.190E−13	0.10002	**2.39004E**−**04**
	EEFO	1.8443	1.9415	1.8325	0.5786	999.986	5.043E−11	4.306E−14	2.078E−11	0.09984	4.73050E−04
	SCSO	1.2214	1.1460	1.9647	0.0000	14.588	0	0	0	0.11376	2.53996E−02
	AVOA	2.0000	1.9999	1.9956	0.0000	70.875	0	3.462E−10	0	0.10129	6.70034E−03
	HHO	2.0000	2.0000	2.0000	0.3489	222.564	1.260E−10	1.260E−10	1.260E−10	0.10042	2.81242E−03
	ZOA	1.0197	1.0539	1.5424	0.0154	43.472	0	0	9.915E−13	0.10318	9.25658E−03
	BDO	1.3690	1.0000	2.0000	0.0000	14.589	0	0	0	0.11376	2.53996E−02

**Fig. 14:**
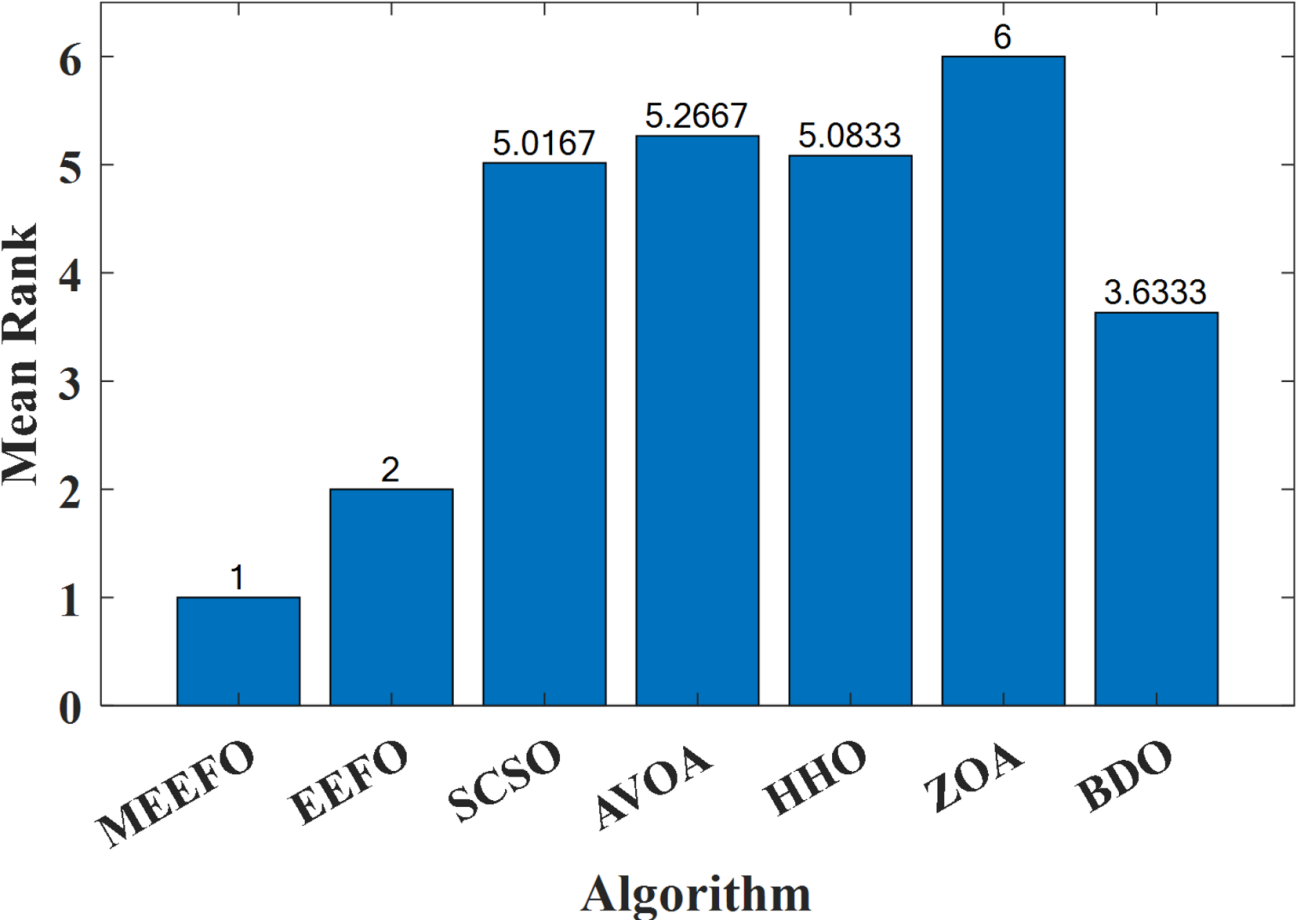
The ranking of the optimizers using the Freiman test for TDM-PVW752.

The statistical evaluations applied to the PV unit cells for RTC France and PVW 752, including the minimum, mean, worst, and standard deviation (Std), are given in Table 8. This table reveals that the suggested MEEFO succeeded with the least Std for the RTC France unit cell, with values of 7.3887E-05 and 2.5637E-04 for the DDM and TDM, respectively, whereas the least standard deviation for the SDM belongs to the BDO algorithm, with a value of 1.1042E-16. For the PVW752 unit cell, the MEEFO algorithm succeeded in achieving the best values for the minimum, mean, worst, and Std for the TDM. Compared with the other introduced algorithms, the proposed MEEFO algorithm is the optimal way to determine the optimal values for various models for the RTC France and PVW752 unit cells. Figure 14 shows the rankings of MMEFO and BDO, the ZOA, SCSO, HHO, and EEFO. The MEEFO method clearly has the lowest mean, and the best performance compared with those of the other comparative methods for TDM-PVW752. The results of Friedman, Kruskal-Wallis and Anova testes are tabulated in Table 9.

**Table 8:** Statistical values of the methods applied to the PV cell.

		Algorithms	mean	min	worst	St.d	p value
RTC	SDM	MEEFO	**0.0013450**	**0.0009861**	**0.0023681**	2.7465E−04	NAN
		EEFO	0.0040755	0.0017932	0.0055354	8.0541E−04	4.0772E−11
		SCSO	0.2095825	0.0153584	0.2228874	5.0588E−02	3.0199E−11
		AVOA	0.2102751	0.0093717	0.2228617	4.8336E−02	3.0199E−11
		HHO	0.0486855	0.0046688	0.1243442	2.9677E−02	3.0199E−11
		ZOA	0.1984049	0.0246525	0.2231808	6.3813E−02	3.0199E−11
		BDO	0.2228614	0.2228614	0.2228614	**1.1042E**−**16**	3.1507E−12
	DDM	MEEFO	**0.0010178**	**0.0009839**	**0.0013072**	**7.3887E**−**05**	NAN
		EEFO	0.0015514	0.0009892	0.0025689	4.2005E−04	1.411E−09
		SCSO	0.0209314	0.0014385	0.0593586	2.0231E−02	3.0199E−11
		AVOA	0.0035923	0.0010625	0.0131908	2.7497E−03	4.5043E−11
		HHO	0.0268900	0.0013972	0.1357264	3.1530E−02	3.0199E−11
		ZOA	0.0029864	0.0010901	0.0058286	1.2005E−03	4.9752E−11
		BDO	0.0299761	0.0010065	0.0460136	1.6194E−02	6.6632E−11
	TMD	MEEFO	**0.0011646**	**0.0009838**	**0.0020724**	**2.5637E**−**04**	NAN
		EEFO	0.0020292	0.0010742	0.0034177	6.0108E−04	4.998E−09
		SCSO	0.0287559	0.0014039	0.2228614	4.0728E−02	6.0658E−11
		AVOA	0.0031009	0.0013466	0.0054699	9.3854E−04	1.0937E−10
		HHO	0.0611490	0.0032646	0.3378319	8.3970E−02	3.0199E−11
		ZOA	0.0042066	0.0012647	0.0097187	2.1340E−03	1.2057E−10
		BDO	0.0253377	0.0009837	0.0434952	1.5797E−02	4.5681E−09
PVW 752	SDM	MEEFO	**0.0003429**	**0.0002886**	**0.0004016**	3.0736E−05	NAN
		EEFO	0.0005083	0.0004484	0.0005773	3.1690E−05	3.0199E−11
		SCSO	0.0248994	0.0103658	0.0254038	2.7449E−03	3.0199E−11
		AVOA	0.0246937	0.0097019	0.0257877	3.0488E−03	3.0199E−11
		HHO	0.0132245	0.0021992	0.0346547	8.8460E−03	3.0199E−11
		ZOA	0.0254160	0.0254025	0.0254500	1.4190E−05	3.0199E−11
		BDO	0.0253996	0.0253996	0.0253996	**3.3477E**−**18**	3.1507E−12
	DDM	MEEFO	**0.0003259**	**0.0002361**	**0.0005228**	7.1762E−05	NAN
		EEFO	0.0005809	0.0004043	0.0007847	8.1354E−05	1.4643E−10
		SCSO	0.0246122	0.0017308	0.0254081	4.3216E−03	3.0199E−11
		AVOA	0.0249022	0.0098914	0.0256464	2.8356E−03	3.0199E−11
		HHO	0.0338904	0.0057233	0.0832857	3.1692E−02	2.9155E−11
		ZOA	0.0254108	0.0253997	0.0254432	**1.2735E**−**05**	3.0199E−11
		BDO	0.0273269	0.0253996	0.0832195	1.0556E−02	4.0988E−12
	TMD	MEEFO	**0.0004169**	**0.0002390**	**0.0005660**	**8.8399E**−**05**	NAN
		EEFO	0.0006222	0.0004731	0.0011167	1.1488E−04	7.3803E−10
		SCSO	0.0292552	0.0253996	0.0832195	1.4669E−02	3.018E−11
		AVOA	0.0247910	0.0067003	0.0255311	3.4169E−03	3.0199E−11
		HHO	0.0398684	0.0028124	0.0832857	3.4630E−02	2.7986E−11
		ZOA	0.0248723	0.0092566	0.0254703	2.9494E−03	3.0199E−11
		BDO	0.0273269	0.0253996	0.0832195	1.0556E−02	6.4328E−12

**Table 9:** The Friedman, Kruskal-Wallis and Anova testes results.

Source	SS	df	MS	Chi-sq	Prob>Chi-sq
Friedman ANOVA results					
Columns	628.383	6	104.731	134.734	1.293E−26
Error	211.117	174	1.213		
Total	839.5	209			
Kruskal-Wallis ANOVA results					
Columns	556883.2	6	92813.86	151.0688	4.60E−30
Error	213550.9	203	1051.975		
Total	770434	209			

Source	SS	df	MS	F	Prob>F
The ANOVA result					
Columns	0.039974	6	0.006662	30.1616	9.67E−26
Error	0.044841	203	0.000221		
Total	0.084815	209			

### PV cells

In the previous section, we discussed how to determine the unknown parameters for various diode models via the suggested MEEFO algorithm for only the simple unit cell of the solar panel. In this section, we study the effect of using the full panel for a larger working area and extract its parameters for the SDM, DDM, and TDM utilizing the MEEFO. Herein, we introduce different types of PV panels, such as STM6-40/36, PWP-201, and STP6-120/36 PV panels. All these PV panels are investigated at 1000 W/m^2^ solar irradiance and different operating temperatures of 45 °C, 51 °C, and 55 °C. The (I‒V) curves for the proposed panels are listed in^66–68^.

**Fig. 15:**
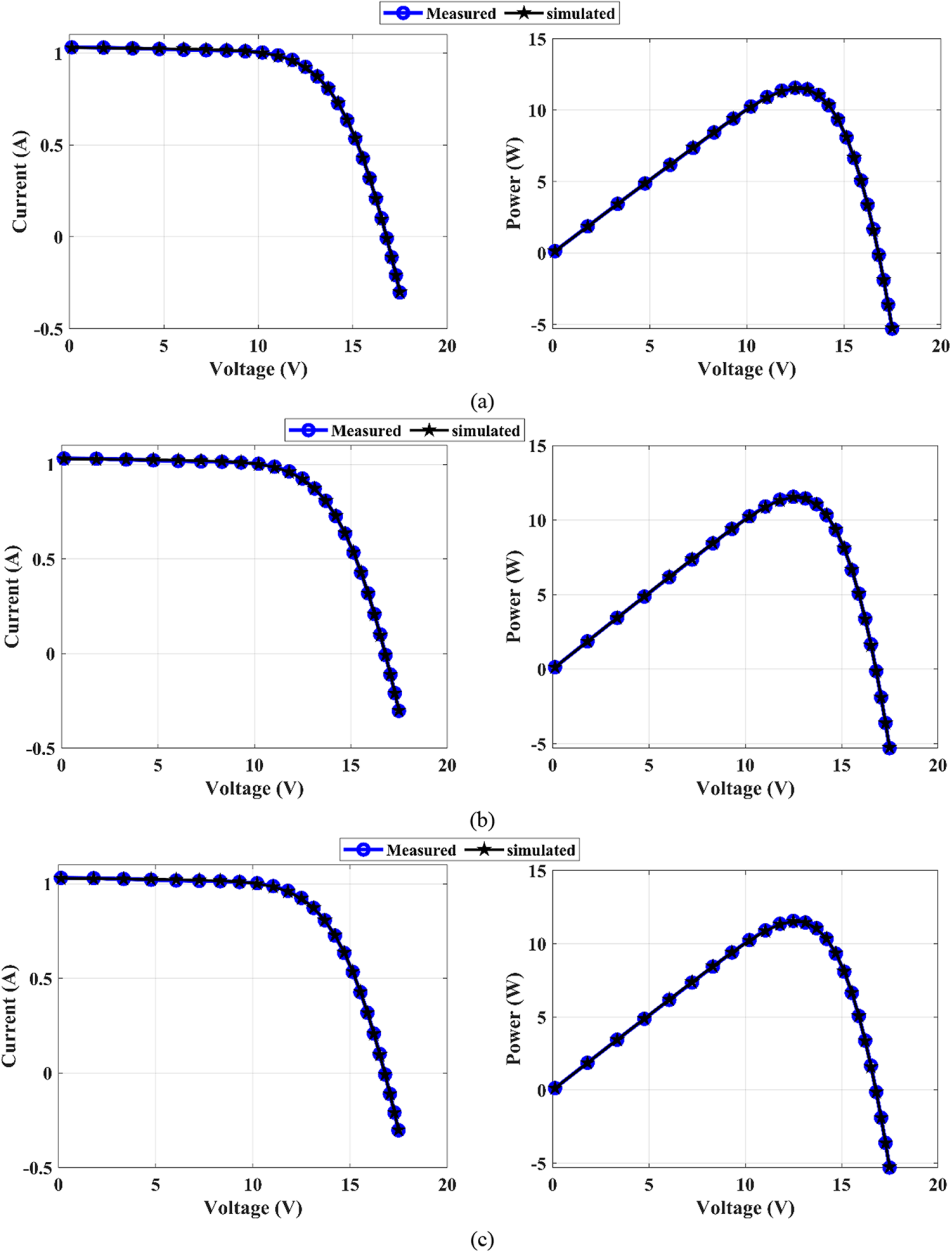
Curves I-V, P-V, I-V, and P-V for PWP-201: (**a**) SDM, (**b**) DDM, and (**c**) TDM

**Fig. 16:**
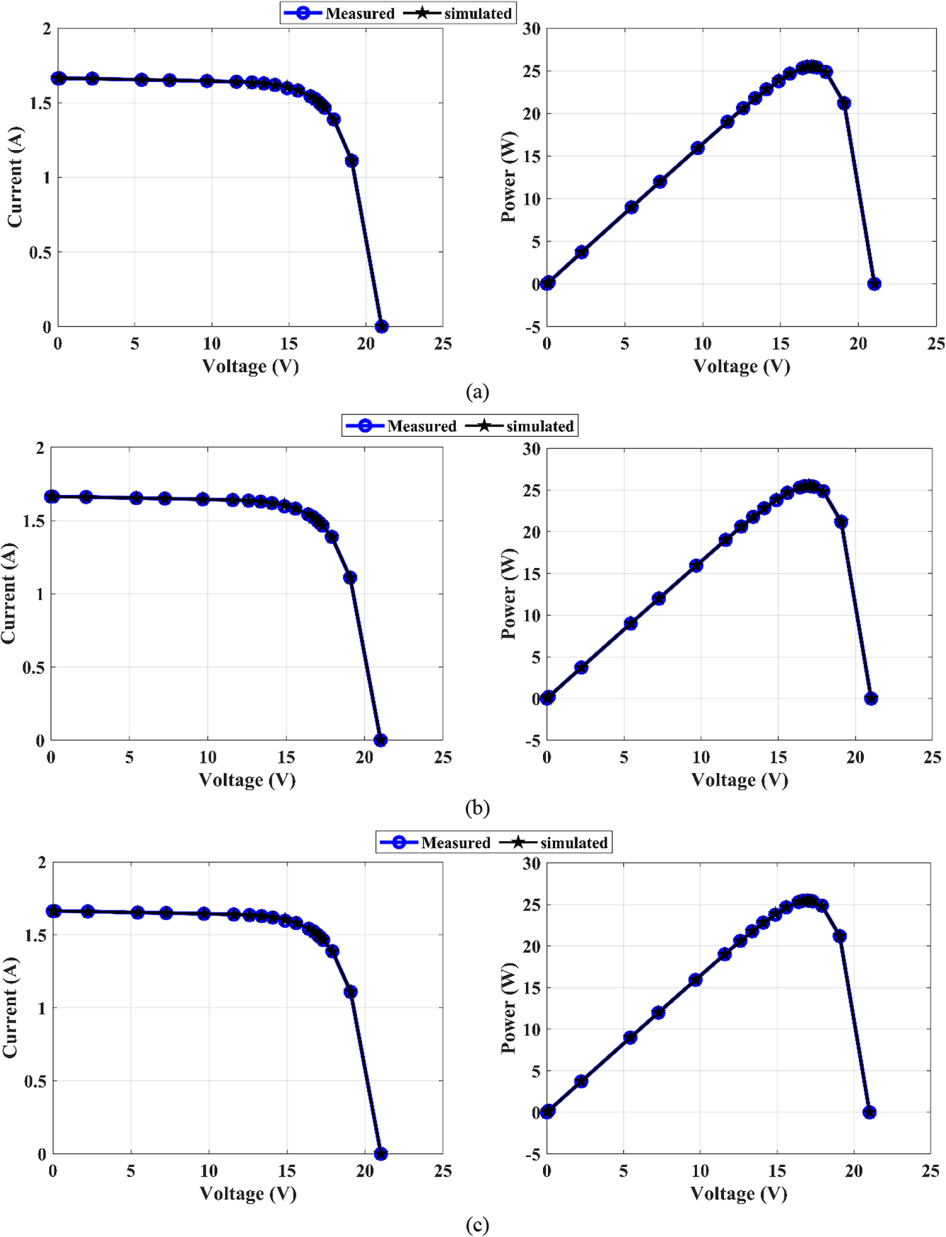
Curves I-V, P-V, I-V, and P-V for PWP-201: (**a**) SDM, (**b**) DDM, and (**c**) TDM

**Fig. 17:**
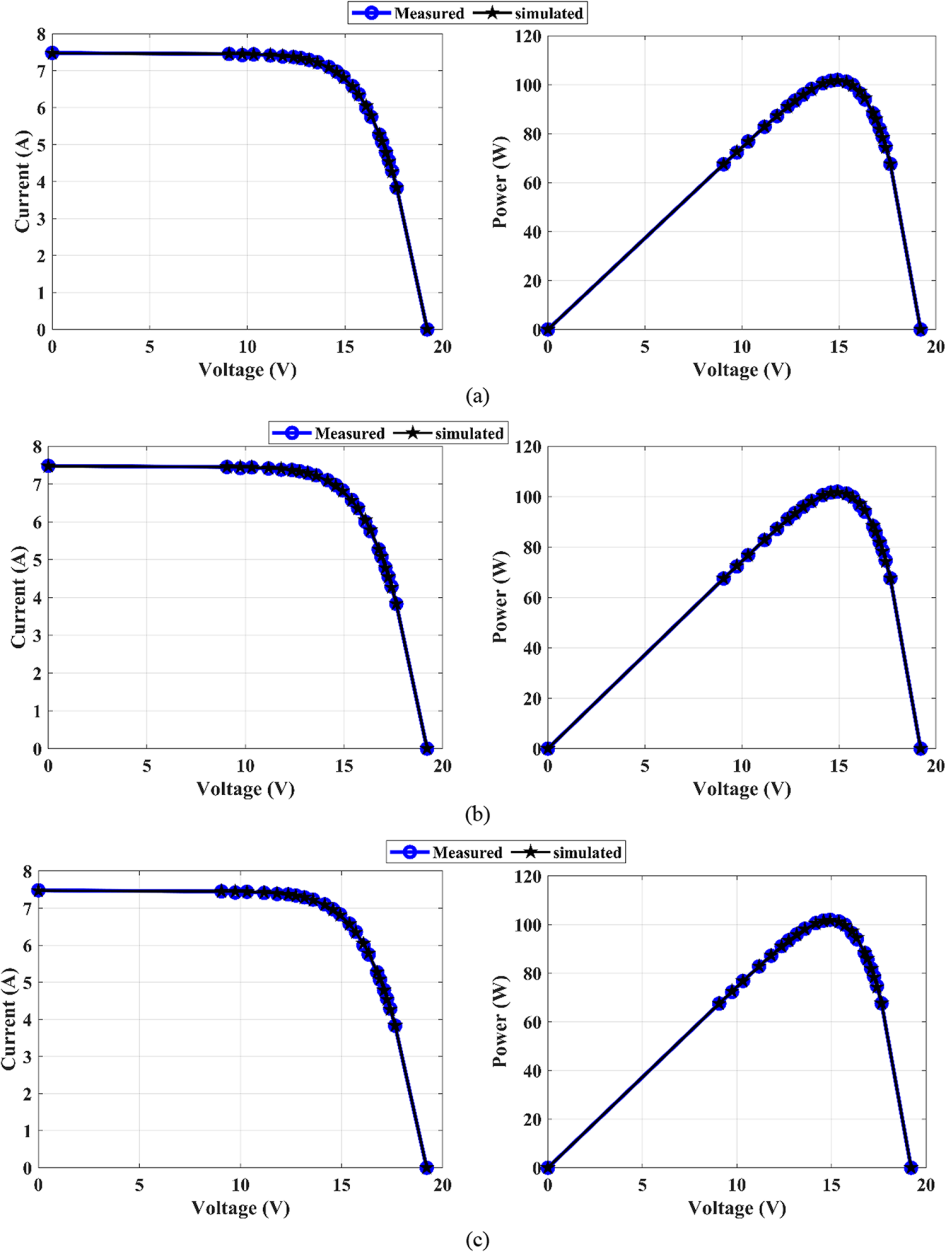
Curves I-V, P-V, I-V, and P-V for PWP-201: (**a**) SDM, (**b**) DDM, and (**c**) TDM

**Fig. 18:**
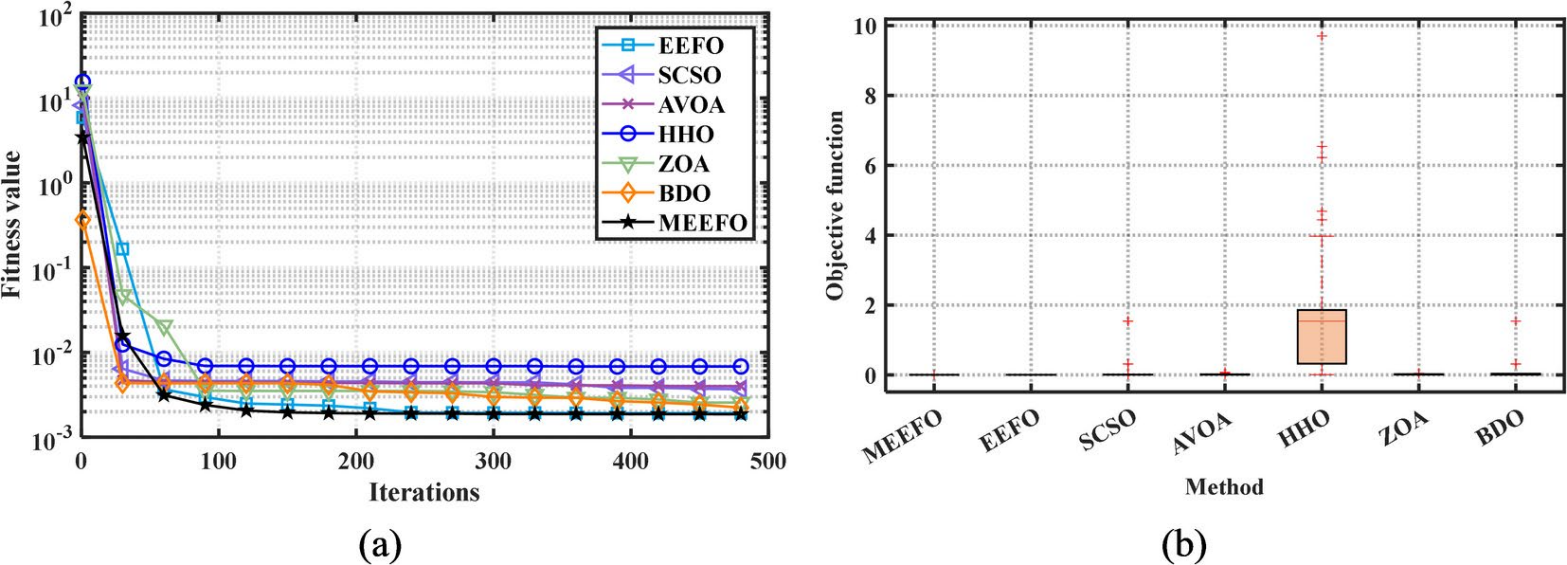
Curves I-V, P-V, I-V, and P-V for PWP-201: (**a**) SDM, (**b**) DDM, and (**c**) TDM

**Fig. 19:**
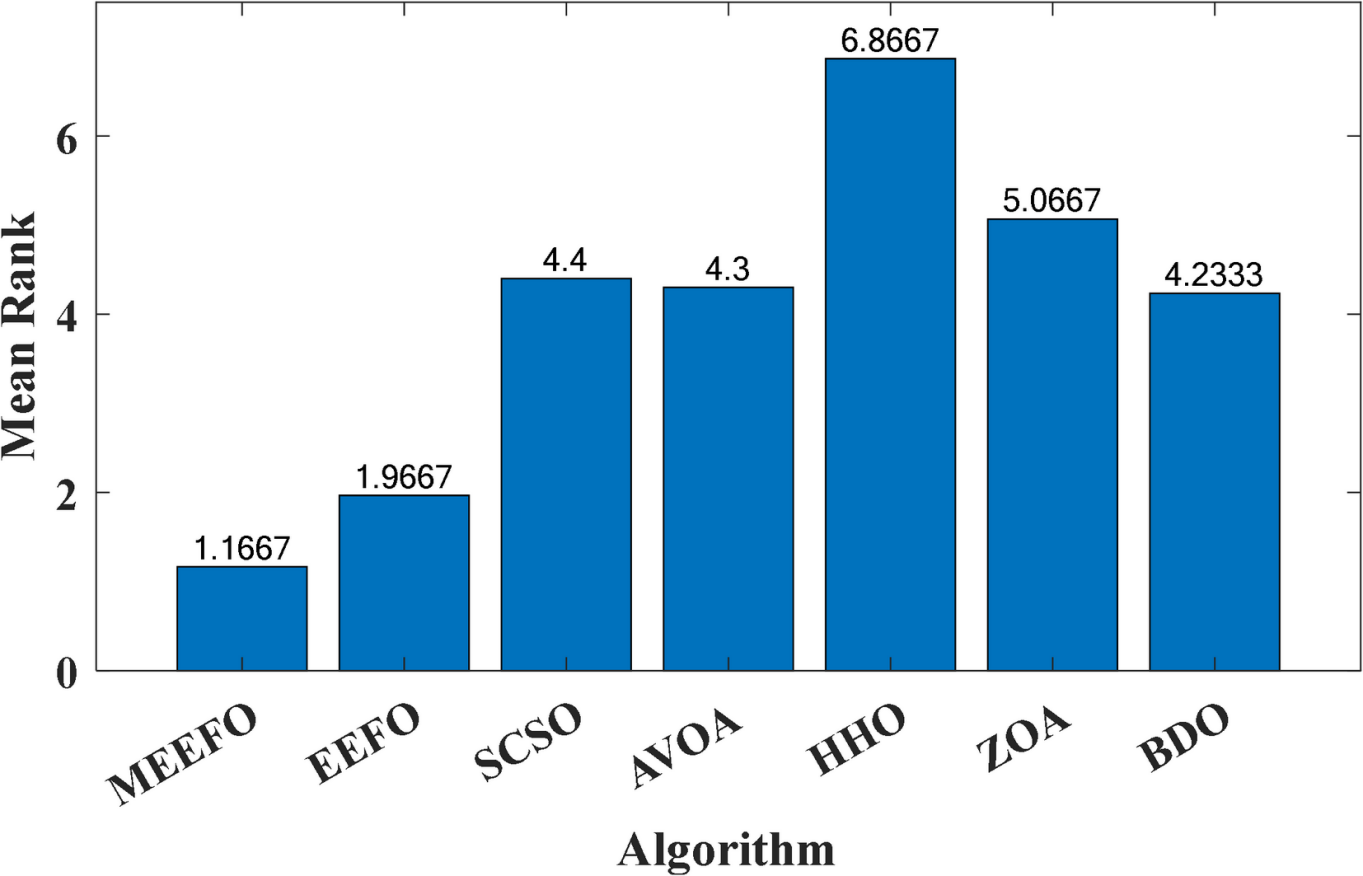
Curves I-V, P-V, I-V, and P-V for PWP-201: (**a**) SDM, (**b**) DDM, and (**c**) TDM

The estimated parameters of the SDM using the MEEFO algorithms in comparison with those of the other introduced algorithms are tabulated in Table 10. This table shows that the lowest RMSE value is attained by the MEEFO for the three proposed solar PV panels of PWP-201, STM6-40/36, and STP6-120/36 PV. The lowest RMSE values are 2.425075E-03, 1.906100E-03, and 1.660060E-02, respectively. The worst value belongs to the HHO algorithms for the PWP-201 solar panel, with a value of 1.435853E-02, whereas the worst values in the STP6-120/36 and STM6-40/36 PVs come from the EEFO algorithm, with values of 1.915104E-03 and 1.660440E-02, respectively. Moreover, Table 11 introduces the DDM’s optimal parameters. The proposed MEEFO yields optimal RMSE values of 2.42511E-03, 1.87603E-03, and 1.66006E-02 for the PWP-201, STM6-40/36, and STP6-120/36 PV panels, respectively. The worst values of the RMSE are those of the HHO algorithm, with values of 4.48130E-03, 9.05503E-03, and 2.64881E-02 for PWP-201, STP6-120/36, and STM6-40/36, respectively. In addition, the TDM’s optimal parameters are given in Table 12, and the optimal value of the RMSE is achieved by the MEEFO algorithm, with a value of 2.42510E–03 for PWP-201 and values of 1.86866E–03 and 1.66089E–02 for STP6–120/36 and STM6–40-/36, respectively. In contrast, the HHO algorithm yields the worst RMSE values of 1.67646E-02, 6.81671E-03, and 6.30669E-02 for PWP-201, STP6-120/36, and STM6-40/36, respectively. Figures 15, 16, and 17 display the (I-V) and (P-V) curves of the proposed MEEFO algorithm for different diode models and different types of PV panels. The measurements and curves align with each other and confirm the effectiveness of MEEFO in the extraction of unknown parameters for PV solar panels. The convergence plots and boxplots for TDM-STM6-40/36 displayed in Figure 18. The results of the statistical analysis are presented in Table 13. This parameter reveals that the proposed MEEFO algorithms succeeded in achieving the best parameter values for different diode models with different types of solar panels. Figure 19 shows the rankings of MMEFO and compared methods. The MEEFO method clearly has the lowest mean, and the best performance compared with those of the other comparative methods for TDM-STM6-40/36. The results of Friedman, Kruskal-Wallis and Anova testes are tabulated in Table 14.

**Table 10:** The optimal parameters for the SDM.

Type	Alg.	α	R_s_	R_sh_	I_d1_	I_pv_	RMSE
PWP-201	MEEFO	48.642898	1.201271	981.9823	3.4823E−06	1.03051	**2.425075E−03**
	EEFO	48.640435	1.201325	980.6285	3.4800E−06	1.03052	2.425076E−03
	SCSO	49.820161	1.169825	1898.9942	4.7088E−06	1.02746	2.607182E−03
	AVOA	48.344781	1.212490	1084.9781	3.2252E−06	1.02943	2.480835E−03
	HHO	46.052181	1.411220	1855.1923	1.7360E−06	1.02939	1.435853E−02
	ZOA	48.771789	1.203396	1508.4321	3.6119E−06	1.02769	2.563464E−03
	BDO	48.561301	1.203450	957.6733	3.4086E−06	1.03065	2.425802E−03
STM6-40/36	MEEFO	53.126973	0.217937	543.7935	1.1469E−06	1.66440	**1.906100E−03**
	EEFO	53.370148	0.210729	559.0847	1.2238E−06	1.66399	1.915104E−03
	SCSO	54.298728	0.204200	623.9969	1.5604E−06	1.66450	2.719815E−03
	AVOA	52.318356	0.242790	524.5112	9.2062E−07	1.66450	1.982593E−03
	HHO	56.659449	0.119242	726.8740	2.7872E−06	1.66267	2.850472E−03
	ZOA	54.512575	0.179531	528.1050	1.6441E−06	1.66656	2.528491E−03
	BDO	54.196093	0.186719	595.6480	1.5194E−06	1.66360	2.015828E−03
STP-120/36	MEEFO	45.363783	0.165407	799.9159	2.3350E−06	7.47253	**1.660060E−02**
	EEFO	45.437881	0.165005	847.6753	2.3930E−06	7.47211	1.660440E−02
	SCSO	47.984602	0.150163	1185.4110	5.3088E−06	7.48703	2.132739E−02
	AVOA	44.676511	0.169724	1380.0470	1.8535E−06	7.46109	1.728181E−02
	HHO	45.080812	0.167333	1399.5418	2.1255E−06	7.46385	1.681243E−02
	ZOA	42.560294	0.180785	399.6230	8.6695E−07	7.46408	2.221771E−02
	BDO	45.551034	0.164500	1500.0000	2.4852E−06	7.46674	1.664312E−02

**Table 11:** The optimal parameters for DDM.

Type	Alg.	α_1_	α_2_	R_s_	R_sh_	I_d1_	I_d2_	I_pv_	RMSE
PWP-201	MEEFO	48.773	48.616	1.2011	983.95	7.078E−07	2.778E−06	1.0305	**2.42511E−03**
	EEFO	48.507	48.623	1.2032	981.81	1.543E−06	1.875E−06	1.0305	2.42715E−03
	SCSO	1.039	50.000	1.1674	1999.47	0.00	4.924E−06	1.0276	2.62965E−03
	AVOA	49.908	43.431	1.1672	1239.24	4.743E−06	9.160E−09	1.0299	2.59355E−03
	HHO	49.005	49.005	1.2285	1240.38	1.910E−06	1.927E−06	1.0306	4.48130E−03
	ZOA	35.310	49.289	1.2252	688.05	2.811E−09	3.707E−06	1.0338	2.72037E−03
	BDO	48.378	1.000	1.2081	885.65	3.247E−06	0.00	1.0311	2.43475E−03
STM6-40/36	MEEFO	48.912	57.784	0.2379	552.00	1.962E−07	1.511E−06	1.6643	**1.87603E−03**
	EEFO	49.221	58.728	0.2248	543.89	2.220E−07	1.791E−06	1.6650	1.93170E−03
	SCSO	50.423	54.345	0.1493	440.58	0.00	1.571E−06	1.6678	3.60756E−03
	AVOA	56.028	59.996	0.0833	1000.00	1.183E−06	2.978E−06	1.6601	3.51633E−03
	HHO	56.343	58.636	0.1950	945.50	1.599E−06	1.657E−06	1.6652	9.05503E−03
	ZOA	58.177	57.918	0.0664	616.18	3.639E−06	2.806E−07	1.6662	4.11959E−03
	BDO	60.000	53.157	0.2170	545.09	0.00	1.156E−06	1.6644	1.90619E−03
STP-120/36	MEEFO	45.356	45.378	0.1654	800.15	1.538E−06	7.966E−07	7.4725	**1.66006E−02**
	EEFO	47.243	44.877	0.1657	1130.53	8.748E−07	1.576E−06	7.4689	1.66483E−02
	SCSO	45.357	24.491	0.1649	638.02	2.328E−06	0.00	7.4734	1.67521E−02
	AVOA	46.339	39.696	0.1602	1500.00	3.208E−06	0.00	7.4746	1.73422E−02
	HHO	44.456	44.520	0.1761	1486.81	9.149E−07	8.153E−07	7.4795	2.64881E−02
	ZOA	43.699	45.015	0.1655	191.07	3.272E−07	1.560E−06	7.5106	2.02957E−02
	BDO	45.630	1.000	0.1640	1126.30	2.550E−06	0.00	7.4702	1.66410E−02

**Table 12:** The optimal parameters for the TDM.

Type	Algo.	α_1_	α_2_	α_3_	R_s_	R_sh_	I_d1_	I_d2_	I_d3_	I_pv_	RMSE
PWP-201	MEEFO	48.594	48.677	48.108	1.2013	982.41	1.195E−06	2.260E−06	2.755E−08	1.03051	**2.42510E−03**
	EEFO	48.239	48.928	48.172	1.2024	973.09	1.346E−06	2.010E−06	1.052E−07	1.03056	2.42589E−03
	SCSO	49.689	17.645	35.987	1.1782	1368.68	4.552E−06	0	0	1.02934	2.60198E−03
	AVOA	46.493	49.984	49.985	1.1649	1816.13	1.723E−09	0	4.898E−06	1.02775	2.61936E−03
	HHO	41.166	38.562	38.562	1.5930	1593.03	5.521E−08	5.521E−08	5.521E−08	1.01786	1.67646E−02
	ZOA	46.847	48.482	47.496	1.1969	549.31	4.419E−07	1.689E−06	7.191E−07	1.03512	3.36094E−03
	BDO	47.576	50.000	50.000	1.2304	752.03	2.619E−06	0	0	1.03209	2.54872E−03
STM6-40/36	MEEFO	49.262	58.755	59.609	0.2372	550.61	2.525E−07	8.094E−08	1.677E−06	1.66449	**1.86866E−03**
	EEFO	56.876	57.203	50.417	0.2418	535.22	4.095E−07	5.181E−07	3.720E−07	1.66448	1.92019E−03
	SCSO	43.350	53.854	8.204	0.2158	506.16	0	1.386E−06	0	1.66792	3.39814E−03
	AVOA	57.836	59.235	59.312	0.0530	1000.00	4.250E−07	1.491E−07	4.328E−06	1.66115	3.99004E−03
	HHO	50.504	50.495	50.547	0.3031	304.14	2.843E−08	4.846E−07	2.843E−08	1.67908	6.81671E−03
	ZOA	55.693	51.813	57.234	0.2259	540.78	5.809E−07	5.078E−07	3.221E−07	1.66601	2.51294E−03
	BDO	55.020	60.000	60.000	0.1636	644.61	1.872E−06	0	0	1.66286	2.22343E−03
STP-120/36	MEEFO	45.379	49.463	45.372	0.1652	940.025	1.313E−06	7.515E−08	1.010E−06	7.47050	**1.66089E−02**
	EEFO	45.823	43.327	48.617	0.1656	886.713	4.932E−07	5.962E−07	1.923E−06	7.47166	1.67527E−02
	SCSO	45.372	1.159	3.261	0.1655	998.423	2.342E−06	0	0	7.47018	1.66173E−02
	AVOA	47.650	48.792	47.427	0.1519	1499.47	1.455E−10	1.274E−06	3.643E−06	7.48266	2.02563E−02
	HHO	37.907	37.888	37.626	0.2019	1407.44	3.907E−08	3.907E−08	3.907E−08	7.39554	6.30669E−02
	ZOA	50.000	50.000	50.000	0.1357	724.590	1.903E−06	3.942E−06	3.597E−06	7.50204	3.00715E−02
	BDO	50.000	45.609	50.000	0.1612	1500.00	1.243E−06	2.200E−06	0	7.47637	1.73616E−02

**Table 13:** Statistical metrics of the approaches applied to PV cells.

		Alg.	mean	min	worst	St.d	p value
PWP-201	SDM	MEEFO	**0.0024254**	**0.0024251**	**0.0024358**	**1.9530E−06**	NAN
		EEFO	0.0024547	**0.0024251**	0.0029513	9.5975E−05	1.77E−10
		SCSO	0.1100467	0.0026072	0.2743372	1.2870E−01	3.01E−11
		AVOA	0.0577214	0.0024808	0.2752580	1.1024E−01	3.019E−11
		HHO	0.1244891	0.0143585	0.4355818	1.0235E−01	3.019E−11
		ZOA	0.0755491	0.0025635	0.2748459	9.7411E−02	3.019E−11
		BDO	0.0785236	0.0024258	0.2742508	1.2135E−01	4.027E−11
	DDM	MEEFO	**0.0024308**	**0.0024251**	**0.0024508**	**7.2064E−06**	NAN
		EEFO	0.0025018	0.0024272	0.0033096	1.6053E−04	1.2541E−07
		SCSO	0.1265274	0.0026296	0.2742795	1.1843E−01	3.019E−11
		AVOA	0.0815728	0.0025935	0.2760970	1.1307E−01	3.019E−11
		HHO	0.3242406	0.0044813	1.0638081	3.1019E−01	3.019E−11
		ZOA	0.1678006	0.0027204	0.2749864	1.1291E−01	3.019E−11
		BDO	0.0806879	0.0024347	0.2742508	1.2062E−01	6.5511E−11
	TMD	MEEFO	**0.0024543**	**0.0024251**	**0.0027562**	**6.6219E−05**	NAN
		EEFO	0.0025460	0.0024259	0.0033224	1.8912E−04	4.639E−05
		SCSO	0.1562649	0.0026020	0.2743169	1.2419E−01	4.0772E−11
		AVOA	0.0680911	0.0026194	0.2744937	9.0094E−02	4.5043E−11
		HHO	1.0368380	0.0167646	11.9413063	2.3827	3.018E−11
		ZOA	0.1187430	0.0033609	0.2748501	1.0139E−01	3.0199E−11
		BDO	0.1009155	0.0025487	0.7839120	1.7232E−01	1.9166E−10
STM6−36/40	SDM	MEEFO	**0.0019086**	**0.0019061**	**0.0019273**	**5.1689E−06**	NAN
		EEFO	0.0021645	0.0019151	0.0030013	2.3569E−04	4.975E−11
		SCSO	0.0477094	0.0027198	0.3109992	1.0508E−01	3.018E−11
		AVOA	0.0047497	0.0019826	0.0134717	2.4465E−03	3.02E−11
		HHO	0.0482279	0.0028505	0.3594900	8.8471E−02	3.02E−11
		ZOA	0.0284585	0.0025285	0.3109828	5.5345E−02	3.02E−11
		BDO	0.0054400	0.0020158	0.0347298	5.5934E−03	2.8448E−11
	DDM	MEEFO	**0.0019350**	**0.0018760**	**0.0020940**	**6.3369E−05**	NAN
		EEFO	0.0026077	0.0019317	0.0037950	4.9051E−04	4.61E−10
		SCSO	0.1089479	0.0036076	0.3109824	1.4538E−01	3.01E−11
		AVOA	0.0094245	0.0035163	0.0414789	9.8235E−03	3.01E−11
		HHO	0.9132388	0.0090550	6.5434718	1.3107	3.01E−11
		ZOA	0.0315142	0.0041196	0.1050591	2.3597E−02	3.019E−11
		BDO	0.0167366	0.0019062	0.3109728	5.6103E−02	1.43E−10
	TMD	MEEFO	**0.0019889**	**0.0018687**	**0.0025135**	**1.6450E−04**	NAN
		EEFO	0.0027427	0.0019202	0.0038438	5.2860E−04	7.12E−09
		SCSO	0.1291384	0.0033981	1.5378420	3.9062E−01	3.01E−11
		AVOA	0.0129980	0.0039900	0.0723607	1.5921E−02	3.02E−11
		HHO	2.0767018	0.0068167	9.7025944	2.2691	3.02E−11
		ZOA	0.0162993	0.0025129	0.0465570	1.0436E−02	3.33E−11
		BDO	0.0829530	0.0022234	1.5378420	2.8532E−01	3.98E−11
STP−120/40	SDM	MEEFO	**0.0166012**	**0.0166006**	**0.0166081**	**1.8138E−06**	NAN
		EEFO	0.0173119	0.0166044	0.0206820	8.3514E−04	3.6897E−11
		SCSO	0.3574904	0.0213274	1.4131220	5.4699E−01	3.0199E−11
		AVOA	0.1666903	0.0172818	1.4131220	4.2264E−01	3.0199E−11
		HHO	0.3978534	0.0168124	5.8655585	1.0565	3.0199E−11
		ZOA	0.2942223	0.0222177	1.4134865	4.2807E−01	3.0199E−11
		BDO	0.0687719	0.0166431	0.3174034	7.9888E−02	3.0123E−11
	DDM	MEEFO	**0.0167097**	**0.0166006**	**0.0181281**	**2.8319E−04**	NAN
		EEFO	0.0192035	0.0166483	0.0258857	2.5043E−03	3.1589E−10
		SCSO	0.4473603	0.0167521	1.4131227	5.6351E−01	6.0658E−11
		AVOA	0.1406806	0.0173422	1.4131220	3.5581E−01	3.3384E−11
		HHO	2.1741865	0.0264881	7.1673174	2.4037	3.0123E−11
		ZOA	0.7839047	0.0202957	1.4142958	5.2477E−01	3.0199E−11
		BDO	0.3204064	0.0166410	1.4131220	4.5924E−01	2.1115E−10
	TMD	MEEFO	**0.0168685**	**0.0166089**	**0.0181181**	**3.9201E−04**	NAN
		EEFO	0.0194383	0.0167527	0.0258827	2.4085E−03	1.2023E−08
		SCSO	0.8026302	0.0166173	6.3775905	1.2109	4.1997E−10
		AVOA	0.0824116	0.0202563	1.4131220	2.5264E−01	3.0199E−11
		HHO	3.2982182	0.0630669	13.6826378	3.6954	3.0123E−11
		ZOA	0.7403001	0.0300715	1.4143930	5.5417E−01	3.0199E−11
		BDO	0.5110653	0.0173616	6.3775905	1.1584	6.9854E−11

**Table 14:** The Friedman, Kruskal-Wallis and Anova testes results for STM6-36/40.

Source	SS	df	MS	Chi-sq	Prob>Chi-sq
Friedman ANOVA results					
Columns	654.6667	6	109.1111	140.2857	8.72E−28
Error	185.3333	174	1.065134		
Total	840	209			
Kruskal-Wallis ANOVA results					
Columns	592936.7	6	98822.78	160.5838	4.45E−32
Error	178771.3	203	880.6469		
Total	771708	209			

Source	SS	df	MS	F	Prob>F
The ANOVA result					
Columns	106.9772	6	17.82954	23.18379	8.90E−21
Error	156.1176	203	0.769052		
Total	263.0948	209			

### PV panel operation in changing weather situations

**Fig. 20:**
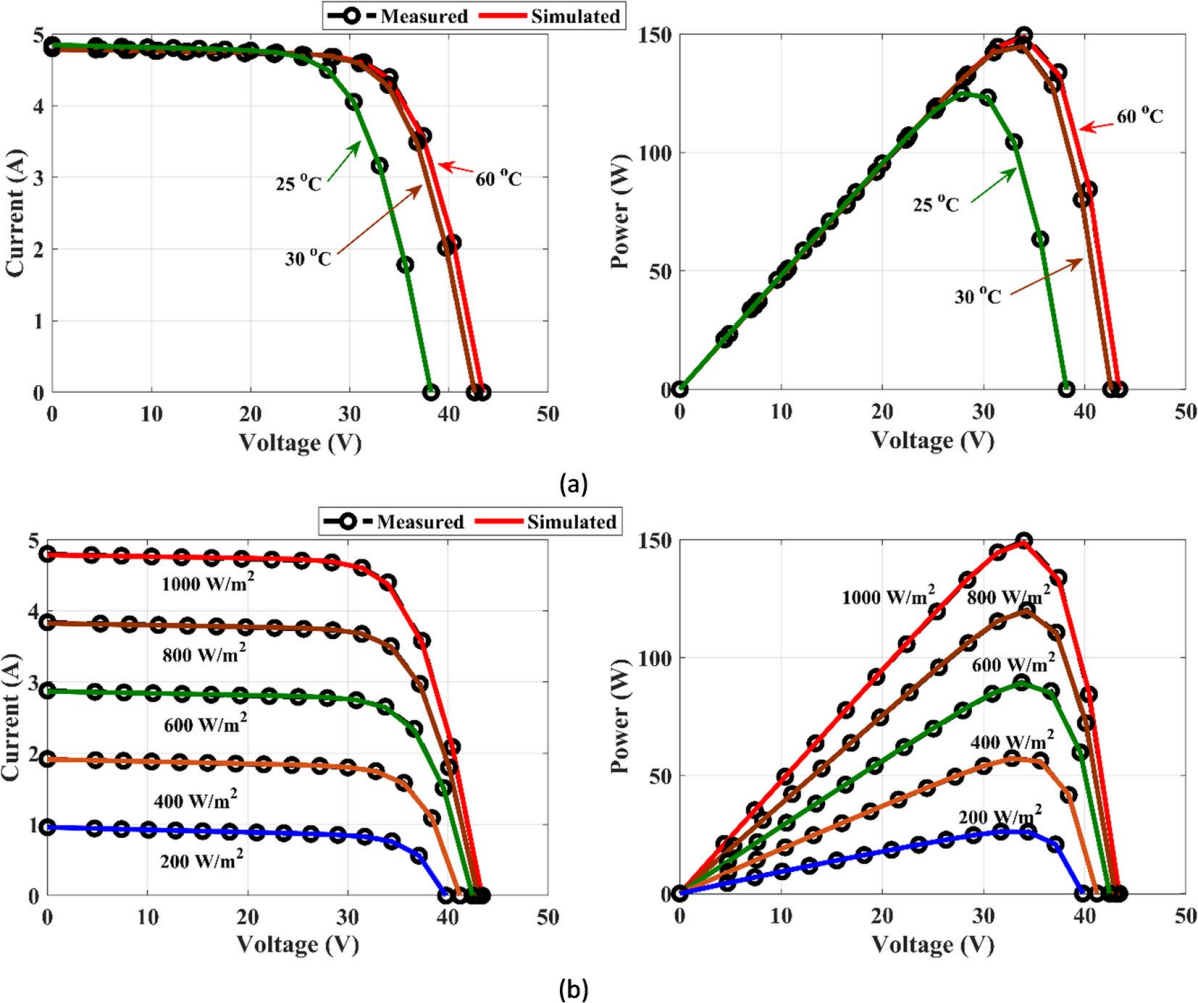
Curves I-V and P-V for SQ150-DDM: (**a**) different temperatures and (**b**) different irradiances.

Temperature fluctuations and solar irradiance variations should be considered when designing PV systems because these factors play a significant role in overall system performance. In this section, MEEFO is applied to establish the DDM of the SQ150 PV panel operating under variable weather conditions throughout the measured data of the current‒voltage relationships presented in^69^. The (I‒V) and (P‒V) curves in Fig. 20a and b of the proposed algorithm of MEEFO illustrate the measured and simulated data for SQ150-DDM at different temperatures (25 °C, 30 °C, and 60 °C) and different solar irradiances (1000 W/m^2^, 800 W/m^2^, 600 W/m^2^, 400 W/m^2^, and 200 W/m^2^). These curves reveal that the simulated data closely match the measured data. Additionally, the statistical metrics of the optimizers applied on SQ150 at various temperatures with constant irradiances of 1000 W/m^2^ are tabulated in the Table 15. This table shows that the optimal temperatures are 30 °C and 60 °C, which results in the lowest standard deviation values of 0.005848 and 0.004331, respectively, throughout the MEEFO algorithm. Furthermore, the statistical metrics of the optimizers applied on SQ120 at various irradiances with a constant temperature of 25 °C are listed in Table 16, which illustrates that the MEEFO algorithm yields the minimum values of the standard deviation compared with those of the other proposed algorithms.

**Table 15:** The statistical metric of the optimizers applied to SQ150 at various temperatures with constant irradiances is 1000 W/m^2^.

T	Alg.	mean	min	worst	St.d	p value
25 °C	MEEFO	**0.023714**	**0.014407**	**0.034261**	0.005899	NAN
	EEFO	0.045911	0.037301	0.056820	**0.005387**	3.02E−11
	SCSO	0.087420	0.043074	0.253778	0.054832	3.02E−11
	AVOA	0.055685	0.041148	0.066353	0.006716	3.02E−11
	HHO	0.264480	0.055786	0.616036	0.182996	3.00E−11
	ZOA	0.057520	0.041616	0.076556	0.007116	3.02E−11
	BDO	0.103582	0.019127	0.167337	0.056947	2.03E−09
30 °C	MEEFO	**0.024647**	**0.012371**	**0.036050**	**0.005848**	NAN
	EEFO	0.044565	0.029624	0.054291	0.007037	1.206E−10
	SCSO	0.090829	0.037378	0.215273	0.053714	3.020E−11
	AVOA	0.055402	0.031851	0.068656	0.007096	4.504E−11
	HHO	0.317258	0.061259	0.625947	0.188604	3.02E−11
	ZOA	0.056404	0.036406	0.065089	0.006039	3.02E−11
	BDO	0.096310	0.017837	0.207450	0.066440	3.08E−08
60 °C	MEEFO	**0.010430**	**0.001339**	**0.021689**	**0.004331**	NAN
	EEFO	0.023999	0.016148	0.033853	0.004697	2.371E−10
	SCSO	0.059132	0.015992	0.285561	0.058741	4.077E−11
	AVOA	0.035318	0.010833	0.059912	0.008500	1.094E−10
	HHO	0.253771	0.039464	0.577071	0.150476	3.020E−11
	ZOA	0.034835	0.020015	0.052136	0.007082	3.690E−11
	BDO	0.067107	0.002835	0.280607	0.091267	3.963E−08

**Table 16:** Statistical metrics of the optimizers applied on SQ120 at various irradiances with a constant temperature of 25 °C.

IRR.	Alg.	mean	min	worst	St.d	p value
800 W/m^2^	MEEFO	**0.020325**	**0.009049**	**0.032090**	0.004878	NAN
	EEFO	0.036167	0.026029	0.042807	**0.004606**	5.49E−11
	SCSO	0.051706	0.022264	0.120577	0.017806	9.91E−11
	AVOA	0.045727	0.029259	0.054174	0.005584	3.33E−11
	HHO	0.213125	0.046040	0.584757	0.130897	3.01E−11
	ZOA	0.047224	0.026524	0.072948	0.012396	4.53E−11
	BDO	0.061467	0.011886	0.094544	0.022426	3.48E−09
600 W/m^2^	MEEFO	**0.014092**	**0.008644**	**0.019908**	0.002768	NAN
	EEFO	0.024607	0.018967	0.029155	**0.002524**	3.68E−11
	SCSO	0.053594	0.021800	0.567598	0.099357	3.01E−11
	AVOA	0.038365	0.018256	0.145491	0.027501	4.07E−11
	HHO	0.160081	0.027780	0.388794	0.097043	3.01E−11
	ZOA	0.042176	0.011008	0.082361	0.018874	6.1E−10
	BDO	0.032473	0.020948	0.034162	0.003065	3.0123E−11
400 W/m^2^	MEEFO	**0.008757**	**0.004880**	**0.013482**	**0.001908**	NAN
	EEFO	0.013066	0.009543	0.016120	0.001522	8.89E−10
	SCSO	0.042994	0.012523	0.370102	0.089094	3.69E−11
	AVOA	0.023696	0.011855	0.036300	0.007477	4.08E−11
	HHO	0.091113	0.014117	0.175673	0.049145	3.02E−11
	ZOA	0.033616	0.011031	0.063773	0.015814	4.07E−11
	BDO	0.027724	0.013863	0.038400	0.007626	2.971E−11
200 W/m^2^	MEEFO	**0.002361**	**0.001350**	**0.003615**	**0.000524**	NAN
	EEFO	0.007003	0.002756	0.012107	0.002726	9.918E−11
	SCSO	0.029496	0.003856	0.171762	0.039008	3.019E−11
	AVOA	0.021117	0.006638	0.030613	0.005753	3.019E−11
	HHO	0.054810	0.019249	0.154299	0.031279	3.019E−11
	ZOA	0.019280	0.003490	0.035525	0.009660	4.077E−11
	BDO	0.018572	0.002758	0.042953	0.012630	3.148E−10

Finally, the MEEFO achieved the best results in most tests and the effectiveness of the algorithm was proven through standard and CEC 2019 functions, but there are some challenges such as the time consumed in the calculation process, in addition to the fact that the proposed method did not achieve the best results in some functions such as CEC- 06, and it gave the same results as other algorithms that were compared for some countries, but in general, the proposed MEEFO proved its efficiency in the tests, especially the evaluation of the optimal parameters of PV.

## Conclusion

A new modified electric eel foraging optimization algorithm with fractional-order calculus, fitness distance balance, and quasi opposition-based learning strategies was presented to designate the PV cell/plate equivalent circuit by calculating its optimum parameters. These techniques improve the capabilities of exploration and exploitation while assisting in avoiding early convergence and local optimization, which are frequently observed in traditional EEFO. Two benchmark suites, the basic and CEC 2019 benchmark equations, are used to evaluate the performance of the proposed MEEFO. Three models of PV. Three different models of PVs were studied to extract their parameters on the basis of their equivalent circuit: the SDM, DDM, and TDM, including the R.T.C. France cell, PVM752 cell, STM6-40/36 plate, PWP-201 plate, and STP6-120/36 plate. by reducing the root mean square error (RMSE) between the simulated and measured currents. The root-meaning square error (RMSE) function is the objective function that represents the difference between the measured and simulated currents. Analyses were conducted on a range of photovoltaic cells and panels under both stable and fluctuating weather conditions. Additionally, many of the comparisons were made with EEFO, SCSO, AVOA, HHO, ZOA, and BDO. The results of the MEEFO approach can be summarized as follows:

- The proposed MEEFO was better than all other optimizers considered for the SDM circuit, obtaining the best RMSE values of 9.861273E-4, 2.886069E-4, 2.425075E-3, 1.9061E-3, and 1.66006E-2 for RTC, PVW752, PWP201, STM6-40/36, and STP-120/36, respectively.
- For DDM, MEEFO produced the best values, with RMSEs of 9.83945E-4 for RTC, 2.36081E-4 for PVW752, 2.42511E-3 for PWP201, 1.87603E-3 for STM6-40/36, and 1.66006E-2 for STP-120/36.
- For TDM, MEEFO realized the lowest RMSE values of 9.8382E-4 for RTC, 2.39004E-4 for PVW752, 2.4251E-3 for PWP201, 1.86866E-3 for STM6-40/36, and 1.66089E-2 for STP-120/36.
- For SQ 150, the best RMSE values at 1000 W/m^2^ were 0.014407 at 25 °C, 0.012371 at 30 °C, and 0.001339 at 60 °C°C, whereas the RMSEs at 25 °C were 0.009049 at 800 W/m^2^, 0.008644 at 600 W/m^2^, 0.004880 at 400 W/m^2^, and 0.001350 at 200 W/m^2^.

The acquired results validated the capability and preference of the suggested MEEFO in creating a dependable comparable circuit for the PV cell/panel operating under various weather circumstances. In the future, a dynamic model of the PV panel should be established. In addition, the parameters of fuel cells and lithium batteries can be extracted to ensure their effectiveness in other systems in the future.

## Electronic supplementary material

Below is the link to the electronic supplementary material.

Supplementary Material 1

## Data Availability

All data generated or analyzed during this study are included in this article.Correspondence and requests for materials should be addressed to G.M.

## Appendix

**Table A1:** Comparison of the MEEFO algorithm's simulation results with various optimizers algorithms (CEC 2019).

Type	MEEFO		IRTHA^70^		KRMG^71^		LCA^72^		FGIN^73^		HFGD^74^		HHO ChaoRand^75^	
Function	Mean	STD	Mean	STD	Mean	STD	Mean	STD	Mean	STD	Mean	STD	Mean	STD
CEC-01	3.6777E+04	1.8074E+03	3.2036E+04	9.8656E+02	0.0000E+00	0.0000E+00	8.2140E+04	1.5000E+04	**1.0000E+10**	0.0000E+00	1.0000E+10	0.0000E+00	4.99E + 04	5.50E + 03
CEC-02	1.7343E+01	7.1360E-15	1.7343E+01	2.7804E-13	1.7340E+01	1.5620E-03	1.7510E+01	6.248E–02	1.7340E+01	1.506E−04	1.7340E+01	1.041E−04	1.7353E+01	7.1500E-03
CEC-03	1.2702E+01	3.6134E-15	1.2702E+00	3.5880E-15	1.2700E+01	4.7030E-07	1.2700E+01	3.645E–15	1.2700E+01	4.767E−14	1.2700E+01	2.208E−14	1.2702E+01	3.4800E-06
CEC-04	2.9351E+01	1.3538E+01	7.0545 E+01	3.6914 E+01	5.2720E+01	1.4600E+01	1.4680E+04	3.4960E+03	4.0470E+01	8.4740E+00	3.8980E+01	6.9370E+00	1.64E + 02	7.3485E+01
CEC-05	1.1199E+00	7.5528E-02	1.1933E+00	1.3940E-01	1.2390E+00	6.461E−02	4.4410E+00	5.932E–01	1.1550E+00	3.562E−02	1.1513E+00	3.263E−04	2.3816E+00	6.1300E-01
CEC-06	8.8030E+00	7.0478E-01	7.4522E+00	1.7572E+00	8.5650E+00	6.502E−01	1.0080E+01	6.086E–01	9.7120E+00	6.852E−01	9.7160E+00	7.416E−01	8.7820E+00	1.1780E+00
CEC-07	6.1789E+01	1.2468E+02	2.2889E+02	1.7654E+02	2.7400E+02	1.7570E+02	7.2290E+02	1.2820E+02	4.3120E+01	7.789E−01	6.3150E+01	5.9410E+01	4.58E + 02	2.61E + 02
CEC-08	3.9969E+00	5.8828E-01	5.1632E+00	5.4960E-01	5.1770E+00	5.3950E-01	6.1440E+00	3.161E–01	**5.3230E+00**	3.660E−01	5.3220E+00	3.845E−01	5.7840E+00	5.1766E-01
CEC-09	2.4229E+00	5.2545E-02	2.4264E+00	1.3168E-01	2.8520E+00	2.0780E-01	2.3100E+03	3.6630E+02	2.6130E+00	1.1250E−01	2.6800E+00	1.379E−01	3.1750E+00	4.4626E-01
CEC-10	1.6502E+01	7.6776E+00	2.0043E+01	6.3500E-02	2.0200E+01	5.4870E-02	2.0420E+01	8.779E–02	2.0290E+01	7.659E−02	1.9940E+01	2.4400E+00	2.0054E+01	7.4995E-02
